# Multiplexed mRNA analysis of brain-derived extracellular vesicles upon experimental stroke in mice reveals increased mRNA content with potential relevance to inflammation and recovery processes

**DOI:** 10.1007/s00018-022-04357-4

**Published:** 2022-05-31

**Authors:** Annika Bub, Santra Brenna, Malik Alawi, Paul Kügler, Yuqi Gui, Oliver Kretz, Hermann Altmeppen, Tim Magnus, Berta Puig

**Affiliations:** 1 Neurology Department, Experimental Research in Stroke and Inflammation (ERSI), University Medical Center Hamburg-Eppendorf, Hamburg, Germany; 2 Bioinformatics Core, University Medical Center Hamburg-Eppendorf, Hamburg, Germany; 3 III Department of Medicine, University Medical Center Hamburg-Eppendorf, Hamburg, Germany; 4 Institute of Neuropathology, University Medical Center Hamburg-Eppendorf, Hamburg, Germany

**Keywords:** Extracellular vesicles, ExRNA, tMCAO, nCounter^®^ panel, Microglia, Oligodendrocytes

## Abstract

Extracellular vesicles (EVs) are lipid bilayer-enclosed structures that represent newly discovered means for cell-to-cell communication as well as promising disease biomarkers and therapeutic tools. Apart from proteins, lipids, and metabolites, EVs can deliver genetic information such as mRNA, eliciting a response in the recipient cells. In the present study, we have analyzed the mRNA content of brain-derived EVs (BDEVs) isolated 72 h after experimental stroke in mice and compared them to controls (shams) using nCounter^®^ Nanostring panels, with or without prior RNA isolation. We found that both panels show similar results when comparing upregulated mRNAs in stroke. Notably, the highest upregulated mRNAs were related to processes of stress and immune system responses, but also to anatomical structure development, cell differentiation, and extracellular matrix organization, thus indicating that regenerative mechanisms already take place at this time-point. The five top overrepresented mRNAs in stroke mice were confirmed by RT-qPCR and, interestingly, found to be full-length. We could reveal that the majority of the mRNA cargo in BDEVs was of microglial origin and predominantly present in small BDEVs (≤ 200 nm in diameter). However, the EV population with the highest increase in the total BDEVs pool at 72 h after stroke was of oligodendrocytic origin. Our study shows that nCounter^®^ panels are a good tool to study mRNA content in tissue-derived EVs as they can be carried out even without previous mRNA isolation, and that the mRNA cargo of BDEVs indicates a possible participation in inflammatory but also recovery processes after stroke.

**Supplementary Information:**

The online version contains supplementary material available at 10.1007/s00018-022-04357-4.

## Introduction

Extracellular vesicles (EVs) are lipid bilayer particles secreted by all types of cells that play an important role in communication among cells as they can transfer proteins, lipids, and genetic material to recipient cells, even over long distances [1, 2]. Among the genetic information contained in EVs, different types of RNA cargoes have been identified such as non-coding RNAs (miRNA, tRNA, siRNA, YRNA, lncRNA, circRNA), and fragmented as well as intact mRNAs [3, 4]. The loading of RNAs into EVs is not random as earlier studies support selective incorporation, reflecting the type and the physiological state of the parent cells [5, 6]. Moreover, several studies have reported that different types of EVs originating from the same cell type contain differentially sorted miRNAs and mRNAs [7–9].

The mRNAs in EVs are transferred to and translated into protein in recipient cells [10–14]. Other types of RNAs such as fragmented non-coding RNAs and miRNAs have been shown to play an important role in cancer development [14–18]. However, many of the physiological consequences of mRNA and non-coding RNAs contained in EVs once taken up by recipient cells are still unclear.

Ischemic stroke is the world-leading cause of sustained disability. It has a complex pathophysiology involving the reaction of both, brain and infiltrating immune cells, the latter due to the breakdown of the blood–brain barrier (BBB) [19]. After the blockage of an artery, a transient lack of glucose and oxygen at the core of the stroke triggers necrotic cell death in neurons which release their content to the extracellular space, generating danger-associated molecular patterns (DAMPs, such as ATP). DAMPs activate microglia and astrocytes, triggering an immune response. Surrounding the ischemic core, there is a hypoperfused area, the penumbra, where cells are still metabolically active and could potentially be rescued within a critical timeframe [20, 21], probably implying a better outcome for the patient [22, 23].

Several studies support the idea that EVs play an important role in deciding the neuronal fate under stress conditions and probably the same applies to the neuronal outcome in the penumbra after stroke. On the one hand, in vitro studies showed that extracellular ATP stimulates microglia to release EVs containing an altered proteome compared to steady-state conditions, triggering an inflammatory phenotype in astrocytes [24]. ATP released by astrocytes also stimulates the release of microglial EVs containing the proinflammatory cytokine IL-1β [25, 26]. On the other hand, EVs derived from non-stimulated astrocytes can rescue neurons under ischemic conditions, a function that depends on the cellular prion protein (PrP^C^) [27]. Furthermore, EVs derived from oligodendrocytes increase the viability of neurons subjected to nutrient deprivation [28–30]. Interestingly, a recent study proposed that neurons release “help-me” signals through EVs [31]. The authors showed that EVs containing miRNA-98 released by neurons were taken up by microglia in vitro and in vivo and, consequently, microglial phagocytosis of stressed but still salvageable neurons was decreased in the penumbra after stroke. Thus, to study the intercellular communication after stroke through EVs is of utmost importance to (i) understand the underlying pathophysiological mechanisms, (ii) identify novel biomarkers, and (iii) develop new therapeutic approaches.

Our present study aimed to investigate if and how the mRNA content in BDEVs changes after stroke compared to sham. To this end, we applied a targeted approach using the nCounter^®^ Neuropathology Panel allowing for the simultaneous assessment of 770 genes related to diverse aspects of neurodegeneration such as neurotransmission, neuron–glia interaction, and neuroinflammation, among others. As these panels can also be used without previous mRNA extraction, another aim was to investigate if this was also applicable to the study of tissue-derived EVs, which would represent a technical advantage as it would eliminate steps in the protocol, thus reducing sample loss and decreasing variability. We subjected mice to transient middle cerebral artery occlusion (tMCAO), a widely established mouse model of stroke, and explored the mRNA content of BDEVs at 72 h after reperfusion, when recovery processes may start to take place. We show that (i) the nCounter^®^ panels can be used for EVs analysis bypassing the mRNA isolation, obtaining similar results to the panels incubated with previously isolated mRNA; (ii) the highest increase in BDEVs from tMCAO mice was observed for mRNAs related to inflammatory and recovery processes with (iii) mRNA top hits being present as full-length and mostly contained in small EVs (≤ 200 nm); (iv) the majority of highly upregulated mRNAs in BDEVs are released by microglia at this time-point even though (v) the most upregulated contributors to the whole BDEV pool at 72 h after stroke are oligodendrocytes.

To the best of our knowledge, this is the first report of mRNA analysis from BDEVs. From a technical point, the present study shows that nCounter^®^ panels are a convenient and reliable method to study EVs as they can be performed without prior mRNA isolation. We also show that full-length mRNAs with possible implications in inflammatory and regenerative processes are increasingly shuttled in EVs after stroke, revealing conceivable regulatory roles in stroke pathophysiology.

## Materials and methods

### Ethics statement

All animal experiments were approved by the local animal care committee (*Behörde für Gesundheit und Verbraucherschutz, Veterinärwesen und Lebensmittelsicherheit* of the *Freie und Hansestadt Hamburg,* project number N045/2018), and performed following the guidelines of the animal facility of the University Medical Center Hamburg-Eppendorf.

Mice used for this study were kept under a 12 h dark–light cycle with ad libitum access to food and water.

### Transient middle cerebral artery occlusion (tMCAO) procedure

12–18-weeks-old male C57BL/6 mice were used for the experiments. Mice were anesthetized and tMCAO was performed as described previously [19]. Briefly, the temporary occlusion of the middle cerebral artery was achieved by inserting a 6.0 nylon filament (602312PK10, Doccol) for 40 min. Control (sham) animals were also anesthetized, and their arteries were exposed but not occluded.

Mice were euthanized 72 h after the tMCAO procedure. Ipsilateral and contralateral hemispheres were stored separately at – 80 °C. For the present study, only the ipsilateral hemispheres were used.

### EVs isolation

EVs were isolated from the ipsilateral hemisphere of mice from the stroke or sham group as described previously [32]. Briefly, frozen brains were slightly thawed in Hibernate E media (Gibco), finely chopped, and digested with 75 U/mL of Collagenase type III (Worthington) in Hibernate E (800 μL/100 mg tissue) for 20 min in a shaking water bath at 37 °C. Digestion was stopped using protease inhibitors (Roche). Samples were centrifuged at 300×*g* for 5 min at 4 °C, the supernatant was collected and further centrifuged at 2000×*g* for 10 min at 4 °C. The resultant supernatant was further centrifuged at 10,000×*g* for 30 min at 4 °C. For some experiments (“filtered samples”, F), the resultant supernatant was passed through a 0.22 µm cellulose-acetate filter (Whatman) or directly used in the next step (“non-filtered samples”, NF). The supernatant was layered on top of a sucrose gradient (2.5 M, 1.3 M, 0.6 M) and centrifuged at 180,000×*g* (corresponding to 31,800 rpm in a SW40Ti rotor) for 3 h at 4 °C. Six fractions were collected, diluted in PBS (Gibco), and further centrifuged at 100,000×*g* (24,000 rpm in SW40Ti rotor) for 1 h at 4 °C. After the initial characterization, fractions 2, 3, and 4 were pooled before dilution in PBS. The pellet was stored at − 20 °C for further RNA isolation or resuspended in RIPA buffer (50 mM Tris–HCl pH = 7.4, 150 mM NaCl, 1% NP40, 0.5% Na-Deoxycholate and 0.1% SDS) or PBS with protease and phosphatase inhibitors (Roche) to be used for western blot or NTA analysis, respectively.

### SDS-PAGE and western blot

EV samples in RIPA were mixed with 4 × NuPage LDS Sample buffer (Invitrogen) and 10 × NuPage Sample reducing agent (Invitrogen) and heated at 70 °C for 10 min. For EVs characterization, the same volume for the 6 fractions was loaded (15 µL) in NuPage 10% Bis–Tris precast gels and run at 150 V. Proteins were then transferred onto nitrocellulose membrane (LI-COR) at 400 mA for 1 h. Total protein content was visualized by staining the membranes with Revert Total Protein Stain Kit (LI-COR), as described by the manufacturer’s instructions. To block unspecific binding, membranes were incubated with 1 × RotiBlock (Roth) in TBST for 1 h at room temperature while shaking and incubated overnight at 4 °C with the following primary antibodies: ADAM10 (1:1,000; EPR5622; Abcam), Alix (1:1,000; #12422–1-AP; Proteintech), CD81 (1:1,000; #10037; Cell Signaling), Flotillin-1 (1:1,000; #610820; BD Biosciences), or GM130 (1:1,000; #61082; BD Biosciences). After incubation, membranes were washed with TBST, incubated for 1 h with the corresponding secondary antibody (1:1,000 Anti-mouse IgG, #7076/Anti-rabbit IgG, #7074; both from Cell Signaling), washed again with TBST, and developed with Super Signal West Femto Substrate (ThermoFisher). The chemiluminescence reaction was detected with a ChemiDoc Imaging Station (BioRad).

For cellular markers’ characterization, 3 µg of proteins measured with the Micro BCA Protein Assay Kit (Thermo Scientific) following the instructions of the supplier, were mixed with 4 × loading buffer (250 mM Tris–HCl, 8% SDS, 40% glycerol, 20% *β*-mercaptoethanol, 0.008% Bromophenol Blue, pH 6.8), boiled for 5 min at 95 °C, loaded onto a 10% Bis/Tris acrylamide gel and subjected to electrophoresis as described above. The following antibodies were used: CD40 (1:500, NB100-56127SS; Novusbio), CNP (1:1,000, C5922; Sigma), EAAT1 (1:500, NB100-1869SS; Novusbio), EAAT2 (1:500, NBP1-20136SS; Novusbio), NCAM (1:1,000, #99746; Cell Signaling), P2Y12 (1:500, #11976-1-AP; Proteintech), PLP (1:1,000, NB100-74503; Novusbio), Synapsin 1 (1:1,000, #106103; Synaptic Systems). After overnight incubation while shaking at 4 °C, the detection was performed as described above.

### RNA isolation

RNA was isolated from the EVs using the Qiagen RNeasy Plus Micro Kit (Qiagen). Briefly, EV pellets from fractions 2, 3, and 4 were resuspended each in 117 µL of Lysis Buffer RLT Plus (Qiagen), vortexed, and pooled together (total of 350 µL as recommended by the supplier) and RNA was isolated following the manufacturer’s instructions. The quality and purity of the isolated RNA were checked using the Agilent 2100 Bioanalyzer following the instructions of the supplier.

### Gene expression analysis with nCounter^®^ panels

Gene expression analysis was performed using the NanoString nCounter^®^ Neuropathology panel (#XT-CSO-MNROP1-12, NanoString Technologies). Two panels were used: one loaded with RNA isolated from BDEVs (that were filtered during the preparation of EVs); and another one where all the samples bypassed the RNA isolation (“non-isolated”, NI) but some were filtered (F) during BDEVs preparation, while others were not (NF).

To run the first panel, 50 ng of previously isolated RNA measured by Qubit™ RNA High Sensitivity Assay Kit (Thermo Fisher) using the 3.0 QuBit Fluorometer, were mixed with RNAse–free water (Qiagen) up to 5 µL. Samples were hybridized for 18 h and mixed with 15 µL of RNAse-free water to be loaded on the nCounter^®^ Sprint Cartridge (#LBL-10038-01, NanoStringTechnologies), following the instructions of the company. The analysis was run for 6 h.

For direct loading of EV lysates (i.e., no previous RNA isolation, NI), frozen EVs were resuspended in RLT lysis buffer and RNAse-free water in a ratio of 1:3 and loaded based on protein concentration measured by Micro BCA Protein Assay Kit (Thermo Scientific). 2.8 μg of proteins were loaded.

### Reverse transcription and quantitative PCR (RT-qPCR)

The cDNA was synthesized from the BDEVs’ isolated RNA using Maxima First-Strand cDNA Synthesis Kit for RT-qPCR (Thermo Scientific), following the supplier’s instructions. The resulting cDNA was loaded in a 1:15 dilution with a master mix (Probe qPCR MM No ROX; Thermo Scientific) with the following TaqMan Probes: Hmox1 (#Mm00516005_m1), Fcrls (#Mm00472833_m1), Cd44 (#Mm01277161_m1), C1qb (#Mm01179619_m1), Gfap (#Mm01253033_m1), all from Thermo Scientific. Asb7 (#Mm01318985_m1) and Fam104a (#Mm01245127_g1), both from Thermo Scientific, were used as housekeeping genes based on the nCounter^®^ analysis. The reaction was performed using LightCycler 96 (Roche) with the following conditions: 50 °C for 120 s, 95 °C for 600 s (10 min), followed by 45 cycles of 95 °C for 10 s, and 60 °C for 30 s and further cooling at 37 °C for 30 s. Samples were run in triplicates. Differential expression was analyzed using the 2^−ΔΔCT^ method. ΔCT was calculated by subtracting the arithmetic means of the CT values of our two housekeeping genes from the CT values of the mRNA of interest. ΔΔCT was calculated by subtracting the average ΔCT of the sham samples from the ΔCT of the stroke samples. The fold change (FC) was calculated as 2^−ΔΔCT^.

### PCR

cDNA was synthesized as above and 5 µL of the resulting EVs cDNA (concentration not measured as the PCR was only intended to be qualitative) or 1 µL of total WT brain cDNA (positive control) or 1 µL of water (negative control) were mixed with 1 × Master Mix: dNTPs 0.4 mM, primers 0.4 µM, Dream Taq polymerase 1 U, 1 × Dream Taq Green Buffer (all from ThermoFischer) and water. The following primers were used: Hmox1: 5′ ATGGAGCGTCCACAGCC 3′ (sense), 3′ GGCATAAATTCCCACTGCCAC 5′ (antisense). C1qa: 5′ ATGGAGACCTCTCAGGGATGG 3′ (sense), 3′ TCAGGCCGAGGGGAAAATGA 5′ (antisense); C1qb: 5′ TGAAGACACAGTGGGGTGAGG 3′ (sense), 3′ TACGCATCCATGTCAGGGAAAA 5′ (antisense); C1qc: 5′ ATGGTCGTTGGACCCAGTTG 3′ (sense), 3′ CTAGTCGGGAAACAGTAGGAAAC 5′ (antisense); Gfap: 5′ ATGGAGCGGAGACGCATCA 3′ (sense), 3′ ACATCACCACGTCCTTGTGC 5′ (antisense); and Cd44: 5′ GTTTTGGTGGCACACAGCTT 3′ (sense), 3′ CAGATTCCGGGTCTCGTCAG 5′ (antisense).

The PCR reaction was performed as follows: 95 °C for 5 min; 95 °C for 45 s and 61 °C for 45 s, and 72 °C for 1 min (× 35 cycles); 72 °C for 5 min. The samples were loaded in a 1.5% Agarose gel mixed with Roti-GelStain (Roth) and run at 120 V for 40 min. The gel was then stained with ethidium bromide (Fluka) and imaged with UVP UVsolo touch (Analytik Jena).

### Nanoparticle tracking analysis (NTA)

Pellets resulting from the EVs isolation were resuspended in 30 µL PBS and 1 µL of this suspension was diluted in PBS at 1:500, and the resulting 500 µL were then loaded into the sample chamber of the LM10 unit (Nanosight, Amesbury, UK). Samples were recorded with ten videos, each 10 s long. Data analysis was performed by NTA 3.0 software (Nanosight) with the following software settings: detection threshold = 6, screen gain = 2. For NTA, only samples that were filtrated through a 0.2 µm filter membrane were measured.

### Transmission electron microscopy (TEM)

For TEM, EV pellets were resuspended in 0.1 M PBS. Drops of these suspensions were placed on parafilm and carbon‐coated copper meshed grids (Plano, Germany) for 5 min for probe adsorption. After 5 min of fixation on drops with 1% glutaraldehyde (Roth, Germany) grids were washed 4 times for 30 s and negatively contrasted using 1% uranyl acetate. Grids were air-dried and analyzed using a Philips CM 100 TEM.

### Analysis of the nCounter^®^ panel data, GO terms enrichment, and cell types of origin

For each of the three datasets, genes with raw expression scores not exceeding the average plus two standard deviations of all corresponding negative control probes, in at least one sham and one stroke sample, were removed from the analysis. Normalization was based on the ten housekeeping mRNAs present on the panel. Differential expression analysis was carried out with DESeq2 [33]. A mRNA was considered differentially expressed if the corresponding absolute log2-fold change (log2FC) ≥ 1 and the False Discovery Rate (FDR) ≤ 0.1.

To perform overrepresentation analysis, clusterProfiler [34] was used in combination with GOslim terms [35].

A gene expression matrix for the data set Whole Cortex & Hippocampus—10X Genomics (2020) was obtained from the Allen Mouse Brain Atlas [36]. To generate this matrix, we used the mRNA values corresponding to log2FCs ≥ 2 and FDRs ≤ 0.1 in the panel with samples I + F.

### Statistical analysis

To analyze the differences between shams and strokes in western blot and NTA, we used GraphPad Prism 8, applying non-parametric Mann–Whitney *U* test in both cases. Statistical significance was considered for *p < 0.05, **p < 0.005, and ***p < 0.001. Values are given as a mean ± standard error of the mean (SEM). The exact *p*-values are given in the main text.

## Results

### Characterization of BDEVs and mRNA isolation

**Fig. 1:**
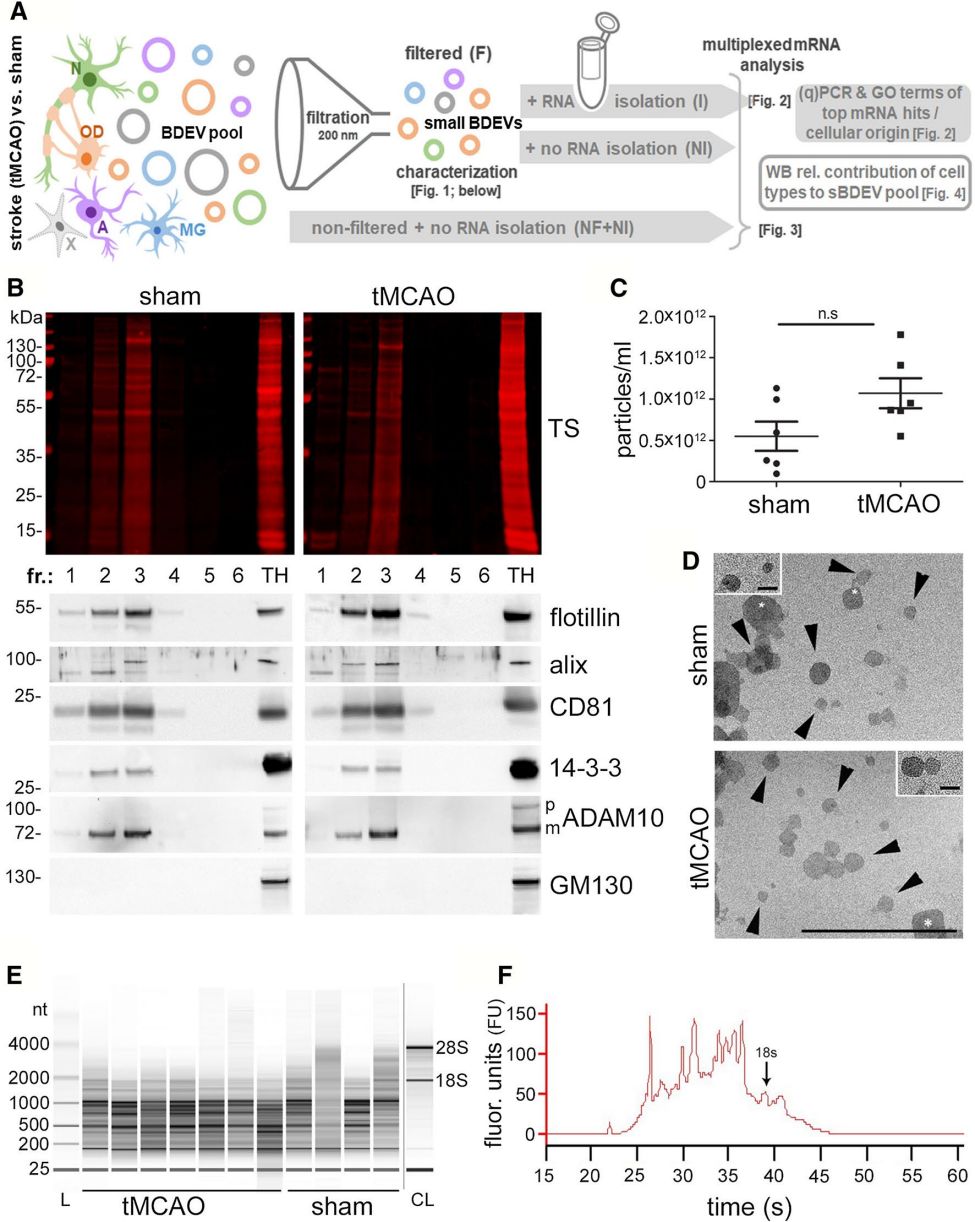
BDEVs characterization and mRNA isolation. **A** Overview of the research strategy and experimental workflow followed in the present study. Different cell types, including neurons (N, green), oligodendrocytes (OD, orange), astrocytes (A, pink), microglia (MG, blue), and others (X, grey) contribute to the EV pool in brain. EVs were purified from the brains of mice 72 h after experimental stroke (tMCAO) or a control procedure (sham). Small brain-derived EVs (sBDEVs) were obtained upon filtration (F) and characterized. The mRNA content of sBDEVs was assessed in detail for stroke and sham samples and compared with (I) or without (NI) a previous RNA isolation step. Moreover, a comparison was performed between filtered (F, sBDEVs) and non-filtered (NF) BDEVs (with the latter population also containing larger EV species). Lastly, changes in the relative contribution of different cell types to the EV pool upon stroke were assessed. Reference to respective figures showing the data is provided in brackets. **B** Western blot characterization of the six fractions obtained after sucrose gradient centrifugation. Fractions 2 and 3 are labeled with antibodies against EV markers flotillin, CD81, and 14–3-3 indicating enrichment of EVs. Moreover, presence in the same fractions of Alix and mature (m) ADAM10 indicates enrichment in exosomes. CD81 and flotillin are also found in fraction 4, therefore, fractions 2, 3, and 4 were pooled for the subsequent experiments. The Golgi protein GM130 is absent in the BDEVs fractions indicating a lack of contamination with intracellular organelles. TS is total protein staining. TH is a total brain homogenate of a WT mouse used only for comparison purposes. **C** Nanoparticle tracking analysis (NTA) of pooled BDEVs fractions (*n* = 6). Values are given in the main text. **D** Electron microscopy of BDEVs. Arrowheads point towards BDEVs, whereas the white asterisks mark structures that, for the shape, are not assignable to EVs and most likely represent some minor contamination by cell membrane fragments. The scale bar is 500 nm, 100 nm on the insert. **E** Example of the BDEVs-RNA profile obtained with the Bioanalyzer. Most of the RNA is under 1000 nt in both, tMCAO and sham BDEVs. CL is a cell lysate, used for comparison purposes as it shows the two main rRNAs (18S and 28S), which are mostly absent in BDEVs. **F** Representative electropherogram obtained with the Bioanalyzer showing the fluorescent units (FU) on the Y-axis and the migration time (in seconds, s) on the X-axis

An overview of our experimental strategy and workflow concerning the respective data in the main figures is shown in Fig. 1A. EVs from mouse brains were isolated as described before [32] and characterized by western blot (WB), nanoparticle tracking analysis (NTA), and electron microscopy (EM) following the MISEV 2018 guidelines [37]. As shown in Fig. 1B, six fractions were obtained after sucrose gradient centrifugation. Fractions 2 and 3 were enriched in common EV markers such as flotillin, CD81, mature ADAM10, 14–3–3, and Alix (with the latter two being cytosolic proteins, indicating EV integrity). GM130, a membrane protein of the cis-Golgi apparatus, was used here as a marker of contamination with intracellular organelles and was not present in any of the six fractions. For subsequent experiments, we pooled fractions 2, 3, and 4 (as in the latter fraction flotillin and CD81 were also present) as the “EV fraction”. NTA measurements of this EV fraction revealed that the number of EVs was not significantly elevated in the tMCAO brains compared to shams, although there was a clear tendency towards an increase (mean value shams: 5.5 × 10^11^ ± 1.77 × 10^11^; mean value tMCAOs: 1.07 × 10^12^ ± 1.82 × 10^11^; Fig. 1C; *n* = 6 for each group). EM pictures showed that EVs of different sizes were enriched in the pooled pellets (Fig. 1D, black arrowheads). Some structures with a more squared profile were also identified (white asterisks) probably indicating some degree of contamination, yet no major differences were observed between EVs isolated from tMCAO brains compared to shams). We then proceeded to isolate RNA from the EV fraction and analyzed it with Bioanalyzer. Most of the RNA contained in the EVs from shams or stroke brains corresponded to small RNAs of less than 1000 nucleotides (nt), although some bands appeared between 1000 and 4000 nt as well. Very low amounts of the ribosomal RNA (rRNA) subunit 18S and no detectable levels of the 28S rRNA subunit were observed (Fig. 1E). A representative RNA electropherogram is shown in Fig. 1F confirming that the majority of RNA is less than 1000 nt with only a few ranging between 1000 and 4000 nt. The mean RNA concentration for shams was 23.06 ± 2.053 ng/µL, whereas for strokes this was 22.06 ± 3.64 ng/µL (*n* = 3 per condition) as measured with QuBit™ RNA High Sensitivity Assay.

### Upregulated mRNAs in BDEVs after tMCAO (i) are related to inflammatory responses, stress defense, and recovery processes, (ii) are present in full-length, and (iii) can be mainly ascribed to microglial origin

Isolated mRNA from EVs purified from the ipsilateral hemisphere of either sham (*n* = 3) or tMCAO (*n* = 3) mice were hybridized with the nCounter^®^ Neuropathology panel from Nanostring which allows for multiplexed detection and quantification of 770 genes related to several aspects of neurodegeneration. Of interest for the studies of EVs’ mRNA, this method can detect low abundant mRNA [38]. To analyze the data, we used two criteria, a stringent one, focusing on those mRNAs that showed an absolute log2 fold-change (log2FC) larger or equal to 2 (i.e., increase/decrease), and broader criteria also considering mRNAs increased/decreased with an absolute log2FC larger or equal to 1, respectively. The false discovery rate (FDR) in each case was required to be below or equal to 0.1.

**Fig. 2:**
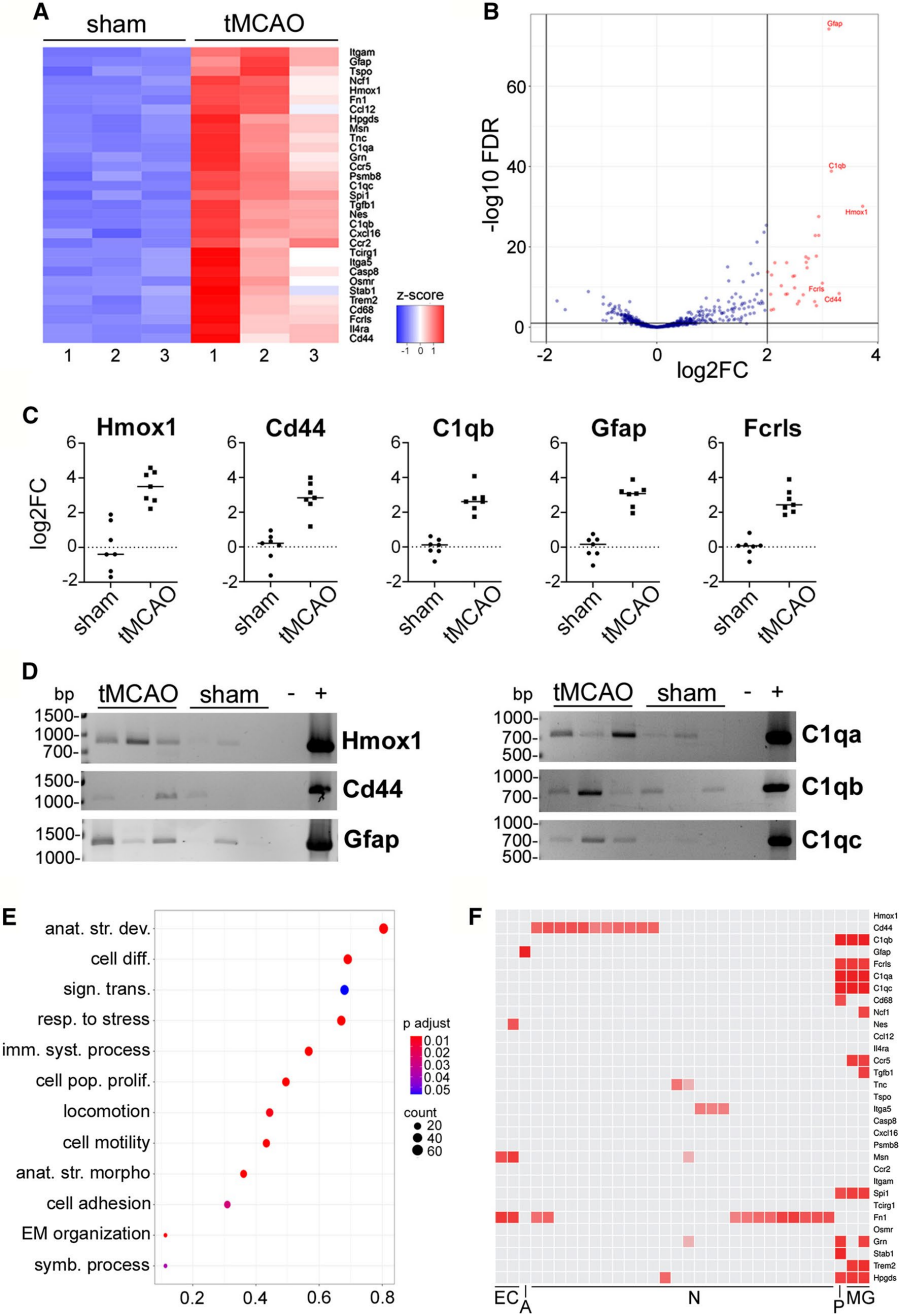
Small BDEVs after tMCAO are enriched in mRNAs related to inflammation, defense response, and recovery processes, with top mRNA hits being full-length and many likely originating from microglia. **A** Heat map showing the significantly up- (log2FC ≥ 2) and downregulated (log2FC ≤ -2) mRNAs found in tMCAO mice compared to shams (*n* = 3 mice per group) using the nCounter^®^ Neuropathology panel with previous mRNA isolation (I) from sBDEVs. **B** Volcano plot for the same results as in (A) displaying the names of the five significantly differentially expressed mRNAs with the highest log2FC (Hmox1: 3.73; Cd44: 3.30; C1qb: 3.16; Gfap: 3.12; Fcrls: 3.00) in sBDEVs from tMCAO mice compared to shams. On the X-axis, the log2FC is plotted while the Y-axis shows the -log10 FDR. **C** Dot plots of the RT-qPCR results of 5 of the top upregulated mRNAs in sBDEVs after tMCAO compared to shams. Every dot represents the log2FC calculated from the ΔΔCT value obtained for each mouse (*n* = 7 per group). **D** Agarose gel images of the PCR products for the six most upregulated mRNAs in sBDEVs from tMCAO brains compared to shams. The results are only qualitative as the cDNA used for the PCR was not measured before the PCR (i.e., the cDNA was added to the reaction by volume). Note that all mRNAs tested present a full-length ORF when compared to the cDNA of wild-type mouse brains used as positive controls (+). (−) is the negative control, where the PCR was performed without cDNA. **E** Enriched GO terms dot plot showing the processes related to the most upregulated sBDEV-derived mRNAs (log2FC ≥ 2) in tMCAO compared to shams. The size of the dots is proportional to the number of mRNAs included in each process, whereas the color indicates the *p adjusted* value. The list and the full names of the GO terms can be found in Table 2. **F** Heat map adapted from the transcriptomics data of the Allen Brain Atlas. *EC* endothelial cells, *A* astrocytes, *N* neurons, *P* perivascular macrophages, *MG* microglia

Out of the 770 genes present in the panel, a log2FC ≥ 2 cutoff resulted in 31 mRNAs showing upregulation with Hmox1 being the highest hit with a log2FC of 3.73 (i.e., about 13-fold upregulated) in the tMCAO BDEVs compared to shams (Fig. 2A and B; Table 1). However, the mRNA with the highest counts significantly upregulated in tMCAO (indicating a strong presence in BDEVs) was for Gfap (Fig. 2B; Table 1). No downregulated mRNA presented a log2FC ≤ − 2. With a cutoff of log2FC ≥ 1, 94 genes were significantly upregulated, and 3 were significantly downregulated (Suppl. Figure 1; Suppl. Table 1). Principal component analysis (PCA) showed that samples of either sham or tMCAO cluster together, respectively, indicating differential mRNA expression between the two experimental groups (Suppl. Fig. 2A).

**Table 1:**
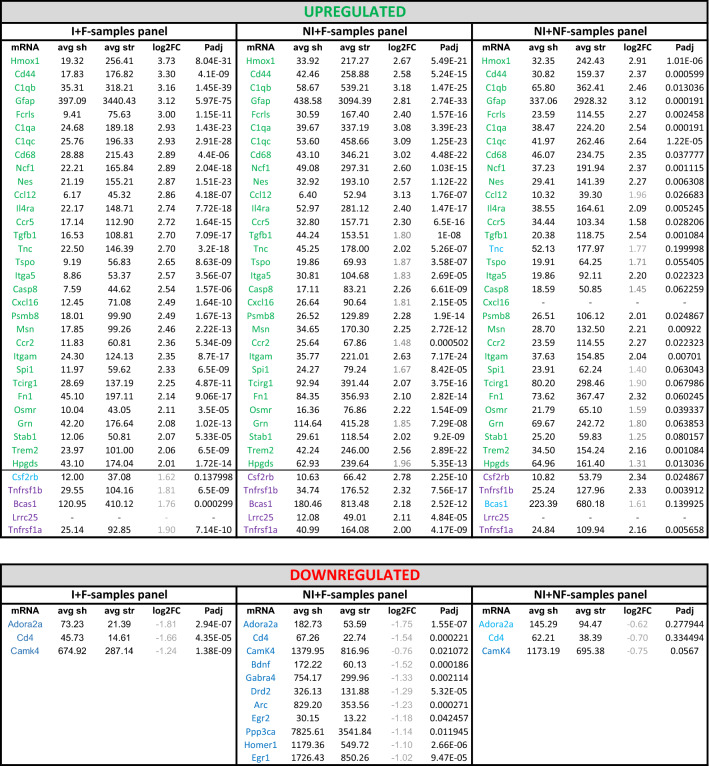
Comparison between upregulated (log2FC ≥ 2) and downregulated mRNAs in isolated vs non-isolated and non-filtered mRNAs from BDEVs in tMCAO compared to shams

Mean counts, and log2FC for each group are provided. Samples with isolated mRNAs and a filtration step (column 1, I + F samples) are taken as the paradigm. The mRNAs of the other samples are compared to column 1 (and therefore, the FC is not in decreasing order). In light grey is the fold change that is less than 2 for the mRNAs than in the I + F samples have a log2FC ≥ 2. In blue, mRNAs that did not satisfy the criteria of FDR ≤ 0.1 but are included in the list for comparison. In violet, mRNAs that were found increased with a log2FC ≥ 2 in the NI + F samples which are then compared with the other two columns. No specific mRNA was found in the NI + NF samples. Downregulated mRNAs are shown in dark blue. None of the downregulated mRNAs was observed with a log2FC ≤ − 2, and therefore, the FC is in grey. CxCl16 and Lrcc25 are not present in all columns as, in these instances, they did not satisfy the premises established for the background

To validate the data obtained with the nCounter^®^ panels, we next performed RT-qPCR for five of the top mRNAs found upregulated in the tMCAO samples. As shown in Fig. 2C, the amount of mRNA extracted from BDEVs was sufficient to perform reverse transcription to cDNA and RT-qPCR analyses (*n* = 7 for each group). All the examined mRNAs (Hmox1, Cd44, C1qb, Gfap, and Fcrls) showed a significant increase in BDEVs from tMCAO mice compared to shams with a similar fold-increase as found in the nCounter^®^ panels (Table 1).

Finally, we designed PCR primers to qualitatively assess whether the mRNA of six of the top ten candidates present in BDEVs was full-length. We used primers allowing for the assessment of the open reading frame (ORF) as an indicator of intact mRNAs. Although the amounts were very variable between the samples, mRNAs for Hmox1, Cd44, Gfap, C1qa, C1qb, and C1qc, were all present in BDEVs with a full-length ORF based on their expected size (Fig. 2D).

The analysis of overrepresentation of GO terms revealed two main types of responses: on the one hand, these were “immune system process” and “response to stress”, indicating that BDEVs carry mRNAs related to inflammatory processes. On the other hand, terms such as “anatomical structure development”, “cell differentiation”, “cell population proliferation”, “anatomical structure formation involved in morphogenesis”, or “extracellular matrix reorganization”, were also overrepresented, suggesting that certain recovery processes are already taking place at 72 h after tMCAO with BDEVs participating in these events (Fig. 2E and Table 2). To assess the likely cellular origin of the highest upregulated mRNAs carried in BDEVs after tMCAO (log2FC ≥ 2), we generated a heat map based on data from the Cell Types Database: RNAseq Data from the Allen Brain Map. This revealed that several mRNAs (13 out of 20 with an assigned cell type) were originating from either microglia or perivascular macrophages (Fig. 2F), although we cannot rule out the possibility that other type of cell types express these mRNAs under ischemic conditions.

**Table 2:** List of GO terms

GO term	
GO:0048856	Anatomical structure development
GO:0006950	Response to stress
GO:0030154	Cell differentiation
GO:0007165	Signal transduction
GO:0002376	Immune system process
GO:0008283	Cell population proliferation
GO:0040011	Locomotion
GO:0048870	Cell motility
GO:0048646	Anatomical structure formation involved in morphogenesis
GO:0007155	Cell adhesion
GO:0030198	Extracellular matrix organization
GO:0044403	Symbiotic process

### Similar BDEVs’ mRNA profiles are obtained with the nCounter^®^ panels with and without previous mRNA extraction, and with and without a filtration step during preparation of BDEVs

**Fig. 3:**
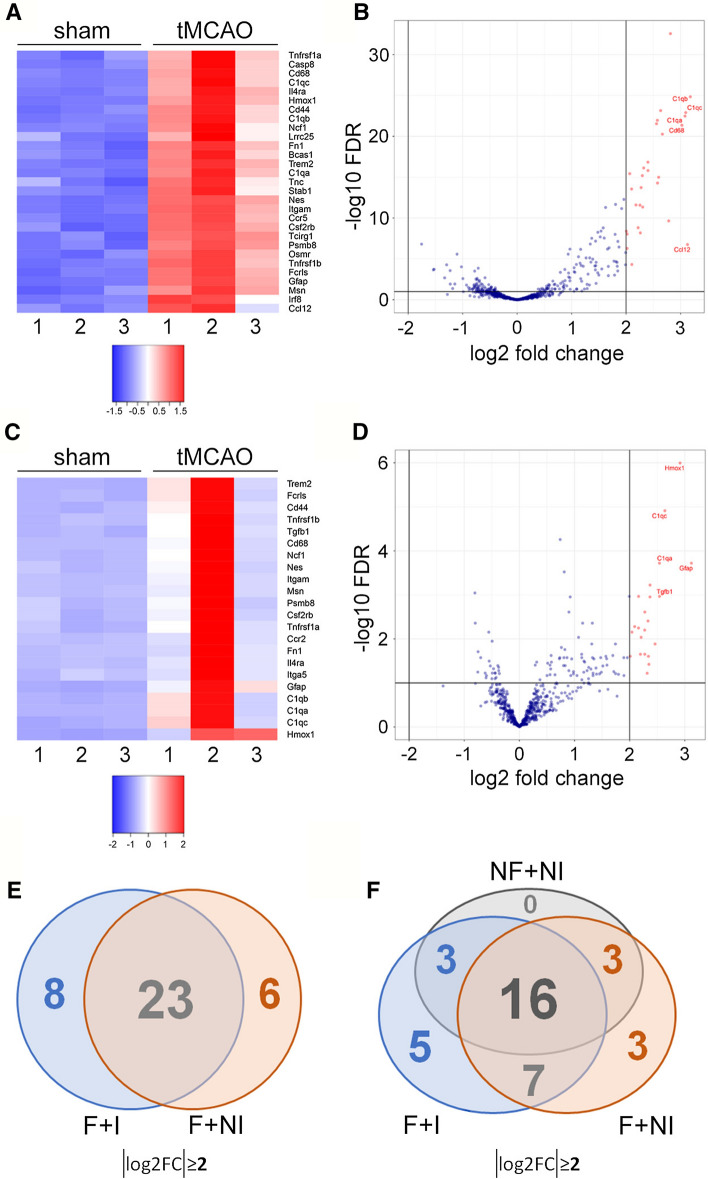
No major differences are observed for the highest upregulated BDEVs mRNAs in tMCAO compared to shams with and without mRNA isolation and with (i.e., sBDEVs) and without a filtration step (i.e., inclusion of BDEVs bigger than 200 µm). **A** Heat map showing the significantly up- and downregulated (absolute log2FC ≥ 2) mRNAs in (filtered) sBDEVs from tMCAO compared to shams (*n* = 3 per group) using the nCounter^®^ panel without mRNA isolation (NI samples). **B** Volcano plot for the same mRNAs as in (**A**) displaying the names of the five highest significantly differentially expressed mRNAs in sBDEVs in tMCAO mice compared to shams. The exact log2FC values for these five mRNAs are C1qb: 3.18; Ccl12: 3.13; C1qc: 3.09; C1qa: 3.08; and Cd68: 3.02. The X-axis shows the log2FC, and the Y-axis the − log10 FDR. **C** Heat map of the most up- and downregulated mRNAs (absolut log2FC ≥ 2) mRNAs in BDEVs from tMCAO compared to shams (*n* = 3 per group) using the nCounter^®^ panel without previous filter step in the BDEV harvest protocol and without RNA isolation (NF + NI). **D** Volcano plot for the same mRNAs as in (**C**) displaying the names of the five highest significantly differentially expressed mRNAs in BDEVs in tMCAO mice compared to shams. The exact log2FC values for these five mRNAs are Gfap: 3.12; Hmox1: 2.91; C1qc: 2.64; Tgfb1: 2.54; and C1qa: 2.54. **E** Overview of the most up- and downregulated mRNAs (absolut log2FC ≥ 2) displayed in a Venn diagram showing the number of the commonly shared versus the unique mRNAs for “F + I” samples (mRNA isolated from sBDEVs; see data in Fig. 2) compared to “F + NI” (mRNA not isolated from sBDEVs before running the nCounter^®^ panel). EV filtration was performed in both instances (i.e., both panels assess mRNAs from sBDEVs). **F** Overview of the most up- and downregulated mRNAs (absolute log2FC ≥ 2) displayed in a Venn diagram showing the number of the commonly shared and the unique mRNAs of each studied condition (including non-filtered EVs without RNA isolation (NF + NI)). F = BDEV samples filtrated during preparation (sBDEVs); NF = BDEVs samples not filtrated during preparation (hence also containing larger EVs); I = mRNA was isolated from BDEVs; NI = mRNA was not isolated before running the nCounter^®^ panel

One of the advantages offered by the nCounter^®^ panels is their sensitivity, as they can be used without prior mRNA extraction. This is intended for cells and, to our knowledge, it has not been employed for EV analyses yet. Considering that EV isolation from tissue already is a multistep process (with the risk of losing material and information at every step of the protocol), we thought it could be of great advantage to bypass the mRNA isolation step, as the latter may result in mRNA loss. Hence, as a proof of concept, we ran a second nCounter^®^ Mouse Neuropathology panel for which the mRNA was not isolated before (from now onwards termed “NI” (non-isolated)) to compare them with the sample of the former panel, where RNA was isolated from BDEVs (from now onwards “I” (isolated) samples). The heat map and volcano plot in Fig. 3A and B show that, with some differences in the fold-change, “I” and “NI” samples shared most of the highly upregulated mRNAs. Thus, out of the significantly upregulated BDEV’s mRNAs with a log2FC ≥ 2 in tMCAO (31 in the “I”-samples panel, and 29 in the “NI” samples panel), 23 were common (Table 1 and Venn diagram in Fig. 3E). Eight mRNAs (Tgfb1, Tspo, Cxcl16, Grn, Ccr2, Hpgds, Itga5, and Spi1) present in the “I” samples panel showed a lower fold-change in the “NI” samples panel, although still showing upregulation (log2FC ≥ 1). Vice-versa, five mRNAs (Lrrc25, Tnfrsf1b, Bcas1, Tnfrsf1a, and Irf8) showed a log2FC ≥ 2 in the “NI” samples, which was lower (although still upregulated with a log2FC ≥ 1) in the “I” samples (Table 1). Heat map and volcano plots of log2FC ≥ 1 “NI” upregulated and downregulated samples (88 mRNAs upregulated with a log2FC ≥ 1 and 11 mRNAs downregulated with log2FC ≤  − 1) are shown in Suppl. Fig. 1 and Suppl. Table 2. With some differences in the fold-change, “I” and “NI” samples also shared most of the differentially expressed mRNAs (76 mRNAs, see Venn diagram in Suppl. Fig. 3A), meaning that 81% of the absolute log2FC ≥ 1 mRNAs significantly differentially expressed in the “I”-samples are likewise significantly differentially expressed in the “NI”-samples. Since differences between the panels could be attributed to panel batches or sample variation in the tMCAO model, we conclude that, overall, the nCounter^®^ panels are suitable for the study of BDEVs mRNA cargo without the need for prior mRNA extraction.

In a previous study, we have shown that introducing a filtration step with a 0.2 µm membrane leads to the differentiation between two EV populations harboring different protein content, with the population with a diameter ≤ 200 µm (i.e., small BDEVs, sBDEVs) being relatively enriched in ribosomal proteins [32]. The results presented above were generated with samples undergoing this filtration step. However, we were also interested in knowing whether the relative mRNAs in BDEV populations would change by skipping this filtration step and including larger EVs as well. Therefore, in the same panel (all “NI”-samples), we also added BDEVs samples that were lacking the 0.2 µm filtration step (“NF” samples) and compared them to the filtrated (“F”) samples described above. As shown in the heat map and the volcano plot (Fig. 3C and D) and Table 1, the most upregulated mRNAs (log2FC ≥ 2) present in the “NF” samples were also among the most upregulated present in the filtrated samples, although the relative fold-change slightly varied. In the “NF + NI” samples, we found 22 mRNAs upregulated with a log2FC ≥ 2, and 60 mRNAs upregulated when the cutoff was set to log2FC ≥ 1. In these samples, no significantly downregulated mRNA species were found with a log2FC ≤ − 1 (Table 1; Suppl. Table 3). As shown in the Venn diagram in Fig. 3D, no mRNA was exclusively detected when the samples were not filtered, not even by applying the less stringent cutoff of log2FC ≥ 1 (Suppl. Fig. 3). These findings could indicate that the majority of mRNAs of the brain EV pool are contained in small EVs (≤ 200 µm). Alternatively, our observations may suggest that, because bigger EVs have a relatively higher protein content, the background of the panels is increased, thereby relatively lowering mRNA detection. This could explain the general low fold-change detection in 2 out of the 3 samples shown in the heat map (Fig. 3C). Of note, both, the “NI” panel and the “NI + NF” panel, show differential sample clusters between shams and tMCAO in the PCA, indicating differential mRNA expression between the two groups (Suppl. Fig. 2B and C).

### Oligodendrocytic EVs are upregulated in the total BDEV pool at 72 h after tMCAO

**Fig. 4:**
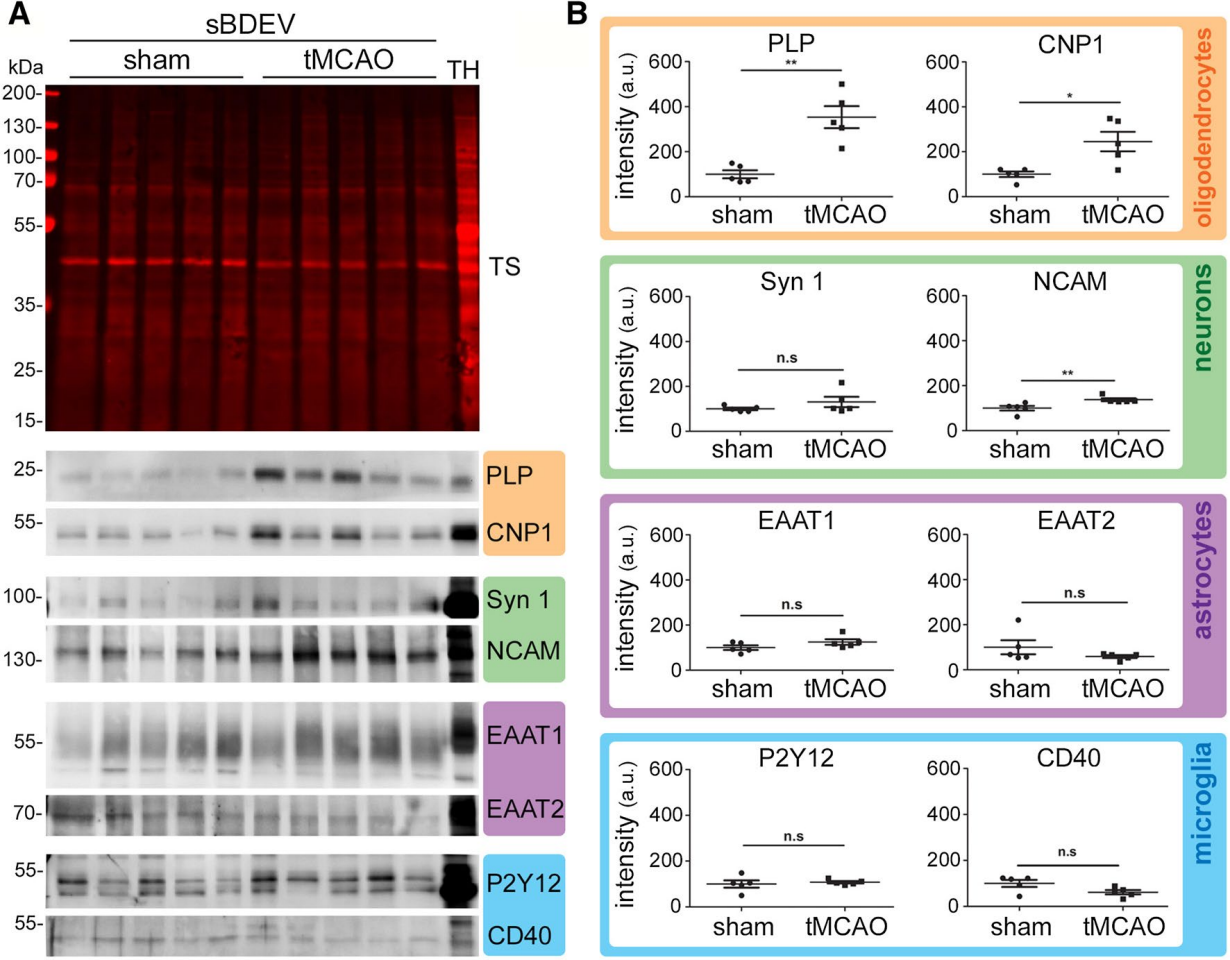
The contribution of oligodendrocytes to the BDEVs pool is significantly upregulated at 72 h after tMCAO. **A** Western blots of sBDEVs samples from tMCAO and shams (*n* = 5 per group) blotted for cell-type-specific markers: PLP and CNP1 are used as protein markers for oligodendrocytes (orange frame); synapsin 1 (Syn1) and NCAM as markers for neurons (green); EAAT1 and EAAT2 as protein markers for astrocytes (pink) and P2Y12 and CD40 as markers for microglia/macrophages (blue). TH is a total mouse brain homogenate loaded in parallel for comparison purposes. TS is a representative total protein staining of the nitrocellulose membranes (TSs of all blots used for these analyses are provided in Suppl. Fig. 5). **B** Dot plots showing the quantifications of the western blot intensities. For the quantification, each band intensity was first referred to the corresponding lanes of the total protein staining. Both markers for oligodendrocytes were found significantly increased upon stroke. Regarding neuronal markers, NCAM was significantly increased while Syn1 only showed a tendency to be elevated. Exact *p-*values are given in the main text

We have demonstrated in a previous study that the main EV population contributing to the total BDEV pool under physiological conditions originated from microglia [32]. This situation changed 24 h after tMCAO when astrocytic EVs were found to be significantly upregulated. Considering our aforementioned finding of a suggested microglial origin of most of the top hit mRNAs identified in BDEVs obtained at 72 h after stroke (Fig. 2F), we wondered whether this would also reflect an increased contribution of this cell type to the total EV pool in stroked brains at this time-point. Therefore, by applying the same methodology as in our previous study (i.e., biochemical assessment of two typical markers per brain cell type), we compared BDEVs from tMCAO (*n* = 5) and sham mice (*n* = 5) at 72 h after stroke. For neurons, we detected the Neural Cell Adhesion Molecule 1 (NCAM1) as it was present in the mass spectroscopy analysis from BDEVs in our previous study, and Synapsin 1 as used before [32]. As astrocyte-specific markers we assessed the Excitatory Amino Acids Transporter 1 and 2 (EAAT1, EAAT2) [39]; and for microglia, we used the G-protein coupled P2Y receptor 12 (P2Y12) as well as CD40, present in activated microglia and macrophages [40]. Lastly, for oligodendrocytes, we detected the proteolipid protein (PLP, a major component of the myelin sheath) and 2′-3′-Cyclicnucleotide 3′-phosphodiesterase (CNP) [41]. The quantification was done by first referring the band intensity of the marker protein to the total protein staining serving as loading control (Suppl. Fig. 5), and then comparing the mean values between sham and tMCAOs. As shown in the western blots of Fig. 4A, all marker proteins were present in BDEVs. An obvious increase in both markers for oligodendrocytes (i.e., PLP and CNP1, significantly upregulated in tMCAO **p = 0.0079 and *p = 0.0317, respectively) was observed (Fig. 4B). This increase was specific for BDEVs as brain homogenates showed no differences between shams and strokes (Suppl. Fig. 4). Surprisingly, although most of the mRNA present in BDEVs in our study is characteristic of microglial origin, both microglial markers used for immunoblotting did not show any increase in the tMCAO samples compared to shams. The neuronal markers NCAM1 and Syn1 were upregulated, but only the increase in NCAM1 reached significance (**p = 0.0079), indicating that neurons could significantly contribute to the BDEVs pool 72 h after stroke. Again, as for PLP and CNP1, the increase in NCAM was specific for BDEVs and not paralleled by a general upregulation in brain homogenates (Suppl. Fig. 4). Finally, although Gfap was one of the mRNAs with highest fold-change in our analysis, we did not observe a significant increase in BDEVs from astrocytes (Fig. 4B). This is in contrast to what we have previously found at 24 h after tMCAO using the same protein markers [32] and may highlight the transient nature of successive inflammatory and repair processes after stroke with the relative contributions from different cell types changing over time.

## Discussion

**Fig. 5:**
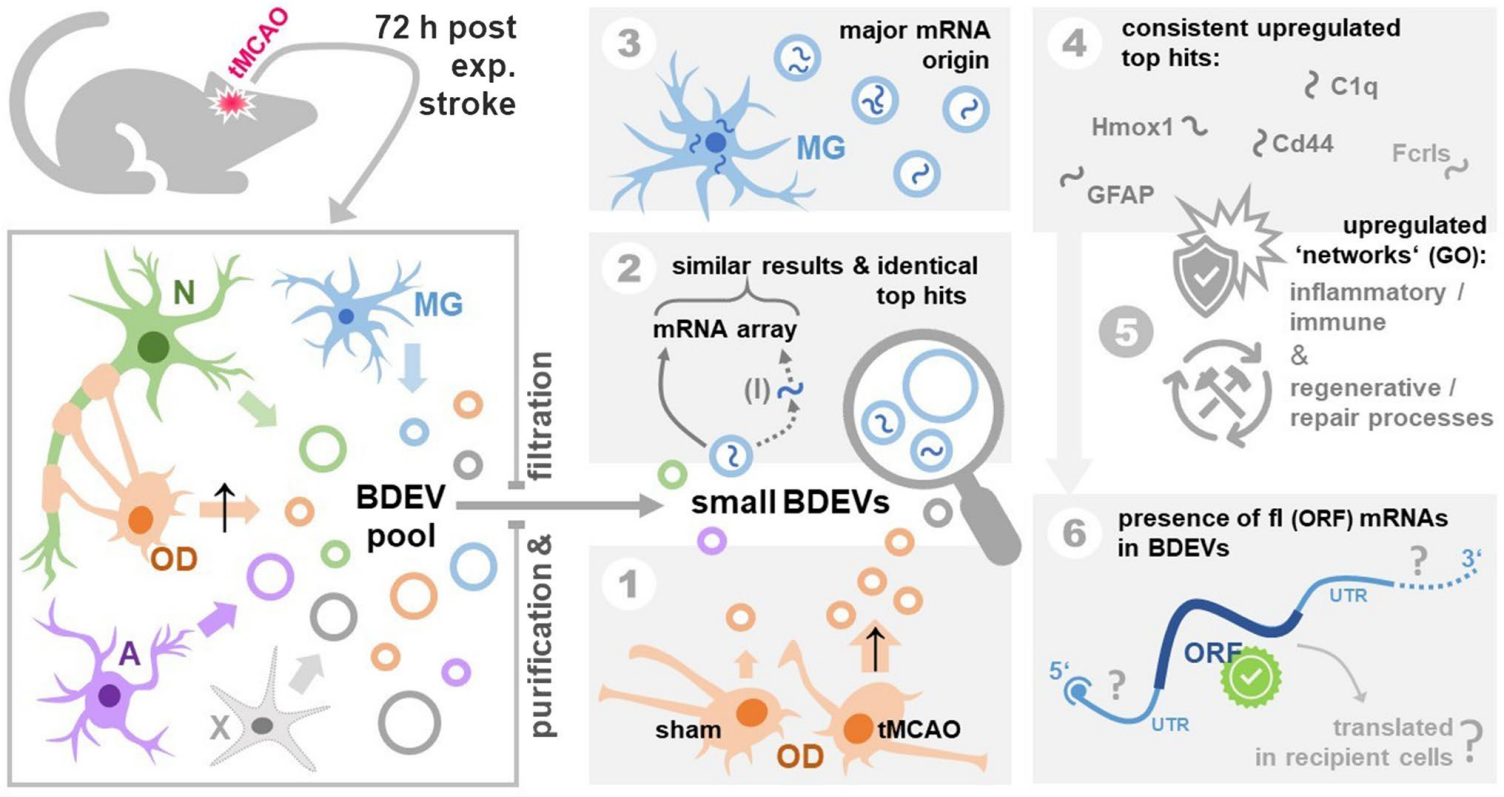
Summarizing scheme of the main findings and open questions. This study assessed alterations in the brain EV pool and the mRNA composition of BDEVs 72 h after experimental stroke (tMCAO) and reperfusion in mice. Neurons (N), microglia (MG), oligodendrocytes (OD), astrocytes (A), and several other cell types (X) contribute to the EV pool in brain (box on the left). Focusing on small BDEVs, we found an upregulated (↑) contribution by ODs to the latter at this time-point after stroke (①). Comparing samples with (I) and without RNA isolation, our multiplexed mRNA arrays revealed similar results regarding mRNA upregulated candidates, and our comparison of filtered and non-filtered BDEVs indicated that mRNAs may be predominantly contained in small BDEVs (②). Though ODs showed an increased contribution to the overall EV pool, mRNAs assessed in BDEVs were mainly of MG origin (③). Consistent top hits showing an increased abundance in BDEVs after stroke included mRNAs for Hmox1, Gfap, Cd44, C1q proteins, and Fcrls (④), and predominant GO terms derived from our mRNA findings could largely be linked with the aspects of inflammatory/immune and regenerative/repair regulation (⑤), thus fitting to processes expected to be initiated and to take place at this time-point after stroke. Notably, we confirmed the presence of ‘intact’ mRNAs (as judged by a full-length (fl) ORF) for 6 top candidates upregulated in BDEVs upon stroke (⑥). Whether these mRNAs also contain important elements such as the 5’ cap structure or the 3’ poly-A tail and, importantly, if these mRNAs are successfully taken up and possibly even translated in recipient cells to elicit (fast) biological responses (e.g., in the context of post-stroke regeneration) remains to be studied further

In the present study (see Fig. 5 for a graphical representation of the main findings), we have used the Nanostring nCounter^®^ panels to demonstrate that, after inducing stroke in mice followed by 72 h reperfusion, BDEVs increased their content in mRNAs related to immune, inflammatory, and defense responses as well as recovery processes. The mRNAs present a full-length ORF in BDEVs, at least for the most upregulated genes in tMCAO compared to shams. The top five most upregulated genes shared by all panels detected here are Hmox1, Cd44, C1q (composed by C1qa, C1qb, and C1qc), Gfap, and Fcrls. Hmox1 encodes for the inducible Heme Oxygenase (HO1), which has been implicated in neuroprotection after stroke and is considered a possible therapeutic target [42]. CD44 protein is important for synaptic plasticity and axon guidance, among other functions, in the central nervous system (CNS). Under pathological conditions such as multiple sclerosis (MS) and its respective experimental model in rodents (Experimental Autoimmune Encephalomyelitis, EAE), CD44 has immunomodulatory properties and protects from the disruption of the BBB [43]. After stroke, CD44 seems to be upregulated in neural stem/progenitor cells (NSPCs) and microglia/macrophages at the penumbra area [44]. C1q, the recognition molecule complex that initiates the classical pathway of the complement cascade, is secreted by macrophages and exerts important functions in the brain such as tagging unwanted synapses for elimination [45, 46]. In stroke, early activation of the complement system leads to inflammation, but at later time-points, C1q might also play a role in regenerative processes [47]. Gfap encodes for the Glial Fibrillary Acidic Protein (GFAP) which is mainly expressed by astrocytes. Its expression increases when astrocytes become reactive and when they form the glial scar which limits inflammation and promotes repair [48–50]. Fcrls encodes for the Fc Receptor-like S, a scavenger receptor expressed specifically by microglia [51] but only in mice, rats, and dogs [52]. Scavenger receptors bind ligands that are non-self or self-altered molecules and remove them by phagocytosis (among other mechanisms) leading to the elimination of degraded or harmful substances [53]. Thus, at 72 h after tMCAO, BDEVs contain mRNAs encoding for proteins involved in inflammatory and defense responses but which may also participate in the reconstruction and repair of the affected area.

Since microglia and astrocytes are reactive and proliferate upon stroke, one question is whether an increase of mRNAs such as Gfap or C1qs in BDEVs is just a consequence of the increased amount of these mRNAs in the parental cells. While with the present study we cannot rule out this possibility (as one would have to assess the amount of mRNA in the cells of origin), the fact that Hmox1, C1q, Cd44, and Fcrls showed rather low counts in general in BDEVs from tMCAO mice (although with a significant fold-increase compared to sham), our findings would speak in favor of a specific loading of these mRNAs into EVs as a reaction to hypoxic ischemia.

It has been shown that different types of EVs contain different RNA profiles, for instance, with apoptotic bodies being richer in ribosomal RNA (rRNA) when compared to microvesicles and exosomes [54]. Interestingly, we did not detect major differences in relative amounts of mRNAs between filtered and non-filtered samples (the latter presenting a larger average diameter [32]). Although we cannot rule out that an increased amount of protein (since the RNA was not isolated, and bigger EVs probably contain a relatively higher amount of proteins) could increase the background (overall fewer mRNAs were detected compared to the “F + NI” panel), it could also imply that sEVs (≤ 200 nm) carry the majority of the EV-associated mRNAs. Crescitelli et al*.* showed that in two out of three investigated cell lines, most of the RNA was present in exosomes (which fall into the category of sEVs) [54]. Although the type of sample and the isolation methods impact the RNA yield and size distribution [55], and though the protocols differ between that and our study, the finding could indicate a general lack (or at least relatively reduced presence) of mRNA in larger EVs. Notably, in our previous study [32] we showed that sBDEVs were enriched in ribosomal proteins when compared to BDEVs that were not filtered and, in fact, ribosomal proteins are commonly found in BDEVs even when using different isolation protocols [56]. Even though it has been shown that EVs do not have the appropriate machinery to translate mRNA directly within the vesicles (at least in cultured cell lines [10]), it could be hypothesized that they could deliver most of the translation machinery plus the mRNA onto recipient cells, particularly as tRNA have also been found in BDEVs [57].

In a previous study, we observed a significant increase in astrocytic BDEVs in the total brain pool of tMCAO mice compared to shams at 24 h after stroke [32]. In the present study investigating the time-point of 72 h after tMCAO, EVs from oligodendrocytes are the most upregulated species as judged by two protein markers in western blot analyses. This came as a surprise considering that much of the BDEVs mRNA that was found to be significantly upregulated at this time-point after tMCAO could be ascribed to microglia. However, given the fact that our analyses only investigated relative changes upon stroke and not absolute contributions by cell types, both findings are not necessarily conflictive as microglia-derived EVs could still make up a large part of the total pool. Of note, oligodendrocytes, the myelinating cells of the CNS, play a currently understudied role in stroke. They are highly susceptible to ischemic conditions, and the remyelination process starts with the proliferation of oligodendrocyte progenitor cells (OPCs) within the penumbra area [58]. Apart from axonal myelination, oligodendrocytes can have an influence on neuronal survival after stroke. For instance, in vitro studies show that upon certain neuronal signals, oligodendrocytes release EVs that rescue primary neurons subjected to oxygen–glucose deprivation (OGD, an in vitro model of stroke) [29, 30]. Oligodendrocyte-derived EVs also influence axonal transport in physiological conditions, an effect that is enhanced when neurons are nutrient-deprived, helping neurons to survive through the delivery of stress-protective macromolecules such as heat shock proteins [28]. Microglia-to-oligodendrocyte communication is another axis that should be considered. Oligodendrocytes can release chemokines and other factors (presumably through EVs) that, under stress conditions, promote microglial phagocytosis [59]. In a model of demyelination in mice, microglia and macrophages turn to anti-inflammatory/immunoregulatory (M2) states and promote the differentiation of OPCs into oligodendrocytes [60]. Thus, by the fact that we also observe an increase in oligodendrocytic EVs at 72 h after tMCAO (a time-point when recovery processes start to take place), it seems plausible that EVs of this particular origin not only play a role in the neuronal remyelination after injury but also in supporting neuronal survival and debris clearance, possibly through communication with microglia.

From a technical point of view, we here present that the Nanostring nCounter^®^ panels are useful tools to study the mRNA composition in BDEVs. We chose this technology because while RNA-seq is a very sensitive method and can detect a wide range of RNAs in a given sample, the nCounter^®^ technology offers the advantage that the starting material can be of low amounts and that no further steps (such as reverse transcription, which can introduce certain biases) are necessary to analyze the mRNA content [61]. We show that even when the RNA was not extracted from BDEVs, similar results (although differing in absolute amounts) were obtained compared to samples that underwent the additional RNA isolation step. This is a clear advantage, as one of the problems when assessing EV-derived mRNA (and a considerable challenge for this field) is the use of different methodologies to isolate EVs’ RNA [62]. Moreover, the approach presented here reduces steps in sample preparation and probably increases the chances to detect some species that otherwise would be lost during mRNA isolation. We were able to detect more significantly downregulated mRNA species when running the panel without RNA isolation, yet overall detection of downregulated mRNAs was rather low. As a possible explanation, we hypothesize that, by the fact that mRNAs are either (i) selectively loaded into EVs (to deliver specific information to recipient cells) or (ii) passively loaded into EVs (due to an overrepresentation/upregulation in the cell of origin), EVs carry specific mRNAs which mostly show an increase over a steady-state situation that may be detected. Moreover, mRNA counts in EVs in steady-state conditions are generally low, and a further decrease after ischemic injury could thus remain undetected due to the panel’s detection limit.

It has recently been described that nCounter^®^ panels are also useful and sufficiently sensitive to analyze EVs isolated from human plasma samples and supernatants of cell cultures. However, in these studies the amount of EVs obtained was too low to run the samples directly, and intermediate steps such as RNA retro-transcription and cDNA amplification had to be introduced [63, 64]. Our demonstration that the panels can be used without previous RNA extraction could help in the search of biomarkers by reducing avoidable extra steps in the protocol and, hence, likely eliminating sources of errors as well as sample and time loss.

Our present report on BDEVs, to the best of our knowledge, is the first to show the suitability of these arrays for analyzing the mRNA content of EVs obtained from complex tissues such as brain.

Some papers described the presence of mRNA in exosomes that could be translated in recipient cells [10, 14, 65], while in other instances mRNA in EVs was found mainly to be fragmented, as in the case of EVs from human glioblastoma stem cells [3, 66]. A recent study using EVs isolated from human blood and performing RNAseq, found that a substantial amount of the total RNA detected in EVs was full-length with a mean size of around 2,800 nucleotides, but also containing very large mRNAs (e.g., KMT2D with more than 19,000 bp) [67]. In our study, we have explicitly checked by PCR if we could amplify the full open reading frame (ORF) of six of the top upregulated candidates. We confirmed that they were all present as full-length in BDEVs, and therefore, have at least the potential to be translated in the recipient cells. However, studying the biological relevance of the mRNA content (and other RNA cargoes) in EVs and proving their functionality in the recipient cells is very challenging [68, 69]. It has been shown in EVs of human glioblastoma stem cells that the number of mRNAs is substantially low, as it was estimated to be 1 copy in 1,000 EVs for the most abundant mRNA, and for others less than 1 copy every 10,000–100,000, or even 1 copy in a million EVs for some RNA species. However, related to miRNA and despite the low numbers, the authors of this study observed an effect of miR21 in recipient cells [3]. We have also made some rough calculations on the possible number of EVs containing mRNA detected in our system: for the NI-samples, we loaded 2.8 µg of sample which approximately corresponds to 3.6 × 10^9^ EVs (calculation based on the mean of 4 NTA measurements and previous micro-BCA assays). If we take Gfap (for which we confirmed presence in full length, and which showed the highest counts among the significantly upregulated BDEV mRNAs in stroke), the ratio would be 1 mRNA copy per roughly 1,000,000 EVs, a similar number of what was previously published [3]. Thus, the biological significance of mRNAs present in BDEVs in these relatively low amounts is difficult to judge with the current knowledge and tools. We have attempted to incubate BDEVs with several cell lines to assess any putative transfer and translation of Gfap and Hmox1 in recipient cells by employing immunocytochemistry and confocal microscopy to no avail (data not shown). However, apart from the low mRNA numbers, several other variables could have played a role in our negative results. In the scenario where EVs would have been indeed delivered and the mRNA translated, it is plausible to think that either confocal microscopy was not sensitive enough to detect newly translated proteins or the time-points we chose to assess protein translation (24 h and 48 h) were not appropriate.

An important question in the EV field is how the cargo is delivered to the cytosol, especially in the case of RNAs that presumably have to reach this compartment in the recipient cell to exert their function/be translated to proteins [68]. Direct fusion of EVs with the target cell’s plasma membrane would be the most plausible mechanism to deliver RNA and the EVs’ luminal content to the cytosol of recipient cells. However, in only a few instances fusion of EVs with recipient cells has been demonstrated until now [70–72], and most of the experiments point to endocytosis as the main uptake mechanism. This implies fusion with late endosomal membranes as a mechanism of endocytic escape for the content of EVs to eventually reach the cytosol [73–75].

In conclusion, we demonstrate here that nCounter^®^ panels are a useful tool for the targeted study of mRNAs contained in EVs derived from brain tissue. Investigations of the EV content and alterations therein after cerebral ischemia will surely contribute to increasing our knowledge of this complex pathophysiology and may provide novel therapeutic tools to rescue neurons at the penumbra soon after hypoxic insults.

## Supplementary Information

Below is the link to the electronic supplementary material.

Supplementary file1 (PDF 1414 KB)

## Data Availability

All relevant data for the present study are in the manuscript and Supplementary Information. Full nCounter^®^ Nanostring panel results, raw and normalized data, are available from the corresponding author upon request.
