# Supplementary material for: Multiplexed mRNA analysis of brain-derived extracellular vesicles upon experimental stroke in mice reveals increased mRNA content with potential relevance to inflammation and recovery processes
Source: Cell Mol Life Sci. 2022 May 31;79(6):329. doi: 10.1007/s00018-022-04357-4 (PMC9156510; doi:10.1007/s00018-022-04357-4)
Supplement: Supplementary file 1 — Supplementary file1 (PDF 1414 KB) [file 18_2022_4357_MOESM1_ESM.pdf]

Multiplexed mRNA analysis of brain-derived extracellular vesicles upon experimental stroke in mice reveals increased mRNA content related to inflammation but also recovery processes

Annika Bub[1]*, Santra Brenna[1]*, Malik Alawi[2], Paul Kügler[1], Yuqi Gui[1], Oliver Kretz[3], Hermann Altmeppen[4], Tim Magnus[1], Berta Puig[1‡]

[1] Neurology Department, Experimental Research in Stroke and Inflammation (ERSI). University Medical Center Hamburg-Eppendorf, Hamburg, Germany.

[2] Bioinformatics Core, University Medical Center Hamburg-Eppendorf, Hamburg, Germany.

[3] III Department of Medicine, University Medical Center Hamburg-Eppendorf, Hamburg, Germany.

[4] Institute of Neuropathology, University Medical Center Hamburg-Eppendorf, Hamburg, Germany.

* shared first authorship

Journal: Cellular and Molecular Life Sciences

Email corresponding author: b.puig-martorell@uke.de

SUPPLEMENTAL TABLES

Suppl. Table 1.- List of the mRNAs found differentially upregulated (log2FC ≥ 1) in BDEVs of tMCAO compared to shams when mRNA was isolated. Downregulated mRNAs are already shown in Table 1.

Suppl. Table 2.- List of the mRNAs found differentially upregulated (log2FC ≥ 1) in BDEVs of tMCAO compared to shams when mRNA was not isolated. Downregulated mRNAs are already shown in Table 1.

Suppl. Table 3.- List of the mRNAs found differentially upregulated (log2FC ≥ 1) in BDEVs of tMCAO compared to shams when mRNA was not isolated and BDEVs were not filtered. Downregulated mRNAs are already shown in Table 1.

SUPPLEMENTAL FIGURES

Suppl. Fig. 1.- Heat maps and volcano plots of the up- and downregulated mRNAs with a log2FC ≥ 1 in all studied conditions. (A) Heat map of the up- and downregulated mRNAs (absolute log2FC ≥1) in BDEVs of tMCAO compared to shams (n=3 mice per group) for the samples where EV preparations were filtered and the RNA was isolated prior to analysis (F+I). (B) Volcano plot for the same mRNAs as in (A) displaying the names of the ten highest significantly differentially expressed mRNAs in BDEVs from tMCAO mice compared to shams. (C) Heat map of the up- and downregulated mRNAS (absolut log2FC ≥1) in BDEVs of tMCAO compared to shams (n=3 mice per group) for the samples where EVs underwent a filtration step during preparation but the RNA was not isolated (F+NI). (D) Volcano plot for the same mRNAs as in (C) displaying the names of the ten highest significantly differentially expressed mRNAs in BDEVs from tMCAO mice compared to shams. (E) Heat map of the up- and downregulated mRNAs (absolute log2FC ≥1) in BDEVs of tMCAO compared to shams (n=3

mice per group) for the samples without EV filtration and RNA isolation (NF+NI) before analysis. (F) Volcano plot for the same mRNAs as in (E) displaying the names of the ten highest significantly differentially expressed mRNAs in BDEVs from tMCAO mice compared to shams. F = BDEV samples were filtrated during preparation (sBDEVs); NF = BDEVs samples were not filtrated during preparation; I = the mRNA was isolated from BDEVs; NI = the mRNA was not isolated before running the nCounter® panel.

Suppl. Fig. 2.- Principal component analysis (PCA) score plots show that BDEVs from shams differentially cluster from BDEVs of tMCAO mice in all three panels. The first two principal components are plotted for each sample. Red circles represent the shams, whereas blue triangles represent tMCAO samples. (A) Panel of filtered EVs and isolated RNA (F+I). (B) Panel of filtered EVs and non-isolated RNA (F+NI). (C) Panel of non-filtered EVs without RNA isolation (NF+NI).

Suppl. Fig. 3.- Venn diagrams showing commonly shared and unique mRNAs for each studied condition with absolut log2FC ≥ 1. (A) Venn diagrams comparing samples where mRNA was isolated with samples lacking RNA isolation, yet EVs underwent a filtration step in both instances (F+I versus F+NI). (B) Venn diagrams comparing the overlap of altered mRNAs (log2FC ≥ 1) found in all conditions investigated herein. F = BDEV samples were filtrated during preparation (sBDEVs); NF = BDEVs samples were not filtrated during preparation; I = mRNA was isolated from BDEVs; NI = mRNA was not isolated before running the nCounter® panel.

Suppl. Fig. 4.- No increase in PLP, CNP1, and NCAM in total brain homogenates from tMCAO mice. (A) Western blots of total brain homogenates from sham and tMCAO samples developed with PLP, CNP1, and NCAM antibodies. TS is total protein staining. (B) Dot plots showing the quantifications for the western blots in (A). n=4 mouse brains per group.

Suppl. Fig. 5.- Total stainings used for the immunoblot quantifications shown in Fig. 4. (A) Total staining (TS) for CD40. (B) TS for P2Y12. (C) TS for synapsin, CNP, and PLP. (D) TS for EEAT1 and NCAM. (E) TS for EAAT2. When several antibodies were used for one membrane, the latter was cut according to the molecular weight of the protein of interest, but never stripped and re-incubated.

Suppl. Table 1

mRNA	avg sh	avg str	log2FC	Padj
Hmox1	19.32	256.41	3.73	8.04E-31
Cd44	17.83	176.82	3.30	4.10E-09
C1qb	35.31	318.21	3.16	1.45E-39
Gfap	397.09	3440.43	3.12	5.97E-75
Fcrls	9.41	75.63	3.00	1.15E-11
C1qa	24.68	189.18	2.93	1.43E-23
C1qc	25.76	196.33	2.93	2.91E-28
Cd68	28.88	215.43	2.89	4.40E-06
Ncf1	22.21	165.84	2.89	2.04E-18
Nes	21.19	155.21	2.87	1.51E-23
Ccl12	6.17	45.32	2.86	4.18E-07
Il4ra	22.17	148.71	2.74	7.72E-18
Ccr5	17.14	112.90	2.72	1.64E-15
Tgfb1	16.53	108.81	2.70	7.09E-17
Tnc	22.50	146.39	2.70	3.20E-18
Tspo	9.19	56.83	2.65	8.63E-09
Itga5	8.86	53.37	2.57	3.56E-07
Casp8	7.59	44.62	2.54	1.57E-06
Cxcl16	12.45	71.08	2.49	1.64E-10
Psmb8	18.01	99.90	2.49	1.67E-13
Msn	17.85	99.26	2.46	2.22E-13
Ccr2	11.83	60.81	2.36	5.34E-09
Itgam	24.30	124.13	2.35	8.70E-17
Spi1	11.97	59.62	2.33	6.50E-09
Tcirg1	28.69	137.19	2.25	4.87E-11
Fn1	45.10	197.11	2.14	9.06E-17
Osmr	10.04	43.05	2.11	3.50E-05
Grn	42.20	176.64	2.08	1.02E-13
Stab1	12.06	50.81	2.07	5.33E-05
Trem2	23.97	101.00	2.06	6.50E-09
Hpgds	43.10	174.04	2.01	1.72E-14
Trf	289.47	1146.01	1.98	4.29E-26
Lox	13.95	54.04	1.96	6.19E-05
Cx3cr1	192.01	739.88	1.94	2.37E-24
Cp	19.72	74.71	1.92	1.08E-06
Eng	16.49	61.07	1.90	3.78E-07
Tnfrsf1a	25.14	92.85	1.90	7.14E-10
Gusb	18.90	69.41	1.88	1.43E-06
Irf8	18.80	67.70	1.84	9.74E-07
Stat3	65.94	233.66	1.83	4.25E-14
Csf1r	94.92	334.07	1.81	6.43E-22
Tnfrsf1b	29.55	104.16	1.81	6.50E-09
Il1r1	32.84	112.62	1.77	1.50E-07
Bcas1	120.95	410.12	1.76	0.000299
Il6ra	18.10	60.08	1.73	9.75E-06
Hexb	138.73	462.43	1.73	1.02E-13
Ccnd1	281.07	913.97	1.70	1.42E-16
Tlr2	11.79	38.04	1.69	0.000444
Csf1	33.73	107.91	1.68	3.77E-07
Hspb1	29.29	92.60	1.66	9.15E-06
Sp100	14.09	43.33	1.60	0.001033
Stat1	40.98	122.48	1.59	1.04E-06
Plcb3	21.17	63.39	1.58	2.22E-05
Nfe2l2	77.10	227.04	1.56	5.03E-12
Notch1	32.72	96.02	1.55	1.20E-06
Col4a1	33.49	96.99	1.54	2.90E-06
Cspg4	13.37	39.04	1.53	0.002946
Cxcr4	10.95	31.38	1.53	0.009501
Tgfbr2	40.57	115.81	1.51	3.34E-08
P2rx7	27.01	77.53	1.51	0.041231
Cd9	27.78	78.57	1.49	2.67E-05
Phf19	10.37	28.86	1.47	0.009891
Gjb1	62.17	167.85	1.43	4.98E-09
Ltbr	27.26	72.64	1.42	1.15E-05
Cdk2	15.77	41.90	1.41	0.009501
Myc	44.15	116.57	1.40	1.57E-06
Mmp14	25.99	68.87	1.40	2.98E-05
Tlr4	11.40	29.77	1.39	0.014332
Cd33	22.77	59.69	1.38	0.000155
Itga7	17.82	45.70	1.37	0.00163
Itpr2	45.28	117.49	1.37	2.22E-05
Gsn	99.13	253.79	1.36	3.80E-08
Plxnb3	48.63	124.19	1.35	4.36E-05
Pecam1	13.69	34.42	1.31	0.012856
Tmem119	50.03	123.79	1.30	4.75E-07
Pcna	200.51	492.34	1.30	3.00E-12
Lif	11.60	28.49	1.29	0.025807
Il10ra	31.24	75.64	1.29	6.19E-05
Pfn1	688.06	1670.34	1.28	9.87E-16
Il13ra1	19.62	46.92	1.27	0.002808
Casp6	28.75	66.22	1.21	0.000247
Pla2g4a	12.96	29.94	1.20	0.022685
Pkn1	44.96	101.68	1.18	0.000325
Col4a2	35.38	80.65	1.17	0.000344
Casp7	19.00	41.40	1.14	0.009778
Sgpl1	56.17	124.47	1.14	7.88E-05
Cdkn1a	76.40	168.00	1.14	1.14E-05
Plekho2	83.98	181.29	1.11	2.18E-07
Nlrp3	14.45	30.21	1.06	0.050228
Arrb2	92.39	190.25	1.04	8.07E-06
Hdac7	85.29	175.53	1.04	0.000166
Myrf	113.40	233.46	1.04	5.00E-05
Fgf2	15.60	31.67	1.02	0.066744
Mapkapk2	44.99	90.50	1.00	0.000828

Suppl.Table 2

mRNA	avg sh	avg str	log2FC	Padj
C1qb	58.67	539.21	3.18	1.47E-25
Ccl12	6.40	52.94	3.13	1.76E-07
C1qc	53.60	458.66	3.09	1.25E-23
C1qa	39.67	337.19	3.08	3.39E-23
Cd68	43.10	346.21	3.02	4.48E-22
Gfap	438.58	3094.39	2.81	2.74E-33
Csf2rb	10.63	66.42	2.78	2.25E-10
Hmox1	33.92	217.27	2.67	5.49E-21
Itgam	35.77	221.01	2.63	7.17E-24
Ncf1	49.08	297.31	2.60	1.03E-15
Cd44	42.46	258.88	2.58	5.24E-15
Nes	32.92	193.10	2.57	1.12E-22
Trem2	42.24	246.00	2.56	2.89E-22
Fcrls	30.59	167.40	2.40	1.57E-16
Il4ra	52.97	281.12	2.40	1.47E-17
Tnfrsf1b	34.74	176.52	2.32	7.56E-17
Irf8	35.74	179.69	2.30	4.55E-12
Ccr5	32.80	157.71	2.30	6.50E-16
Psmb8	26.52	129.89	2.28	1.90E-14
Casp8	17.11	83.21	2.26	6.61E-09
Msn	34.65	170.30	2.25	2.72E-12
Osmr	16.36	76.86	2.22	1.54E-09
Bcas1	180.46	813.48	2.18	2.52E-12
Lrrc25	12.08	49.01	2.11	4.84E-05
Fn1	84.35	356.93	2.10	2.82E-14
Tcirg1	92.94	391.44	2.07	3.75E-16
Stab1	29.61	118.54	2.02	9.20E-09
Tnc	45.25	178.00	2.02	5.26E-07
Tnfrsf1a	40.99	164.08	2.00	4.17E-09
Nlrp3	12.80	49.83	1.99	1.69E-06
Slc11a1	9.04	36.79	1.99	5.25E-05
Hpgds	62.93	239.64	1.96	5.35E-13
Cx3cr1	409.28	1547.84	1.92	7.01E-12
Tspo	19.86	69.93	1.87	3.58E-07
Grn	114.64	415.28	1.85	7.29E-08
Cybb	9.90	37.26	1.85	0.000303
Tlr2	11.68	41.08	1.84	7.97E-05
Itga5	30.81	104.68	1.83	2.69E-05
Cxcl16	26.64	90.64	1.81	2.15E-05
Csf1r	244.52	851.83	1.81	2.16E-12
Tgfb1	44.24	153.51	1.80	1.00E-08
Icam1	9.43	32.81	1.79	0.000971
Cdk2	22.61	78.32	1.78	1.90E-05
Il10ra	51.64	168.11	1.69	4.44E-07
Hspb1	43.41	139.81	1.67	0.001139
Spi1	24.27	79.24	1.67	8.42E-05
Cspg4	32.60	101.55	1.66	6.37E-07
Hexb	290.29	892.95	1.62	5.12E-12
Csf1	73.20	221.98	1.60	2.82E-08
Trf	641.55	1918.99	1.58	1.62E-09
Sp100	24.91	72.37	1.55	0.000173
Myc	64.75	187.60	1.54	4.00E-09
Cd9	53.16	152.56	1.53	2.06E-08
Lif	14.76	41.76	1.50	0.000949
Ccnd1	612.13	1712.53	1.48	5.58E-09
Ccr2	25.64	67.86	1.48	0.000502
Tgfbr2	56.67	155.89	1.45	2.67E-05
Tnf	10.49	29.53	1.45	0.010063
Ltbr	34.57	97.37	1.44	6.77E-05
Nfe2l2	149.76	406.26	1.44	6.81E-08
Tnfrsf12a	24.87	68.94	1.44	0.00022
Cd33	36.76	97.68	1.43	7.46E-05
Cp	36.54	94.97	1.39	1.08E-05
Gjb1	107.80	275.98	1.37	3.01E-08
P2rx7	66.87	170.64	1.35	2.52E-05
Gsn	132.49	336.21	1.34	1.53E-07
Tmem119	94.77	237.29	1.33	7.58E-06
Epha2	11.94	29.14	1.33	0.015798
Plcb2	29.12	73.57	1.30	0.000737
Gpr84	9.84	23.14	1.29	0.043378
Tlr4	20.42	50.44	1.28	0.013009
Stat3	158.14	380.69	1.27	7.60E-06
Casp7	26.94	64.04	1.23	0.0496
Gusb	57.42	133.49	1.21	7.97E-05
Tnfrsf10b	10.63	22.55	1.19	0.086192
Psmb9	23.98	54.63	1.18	0.005509
Plcb3	51.73	118.85	1.17	6.39E-05
Eng	53.29	116.38	1.17	0.002662
Il1r1	76.98	171.10	1.17	6.88E-07
Stat1	86.53	191.90	1.16	3.38E-05
Itpr2	132.97	288.19	1.13	5.51E-06
Phf19	16.22	34.71	1.09	0.047613
Casp1	12.67	26.97	1.09	0.053016
Notch1	125.95	266.27	1.09	4.81E-05
Il6ra	45.97	96.91	1.06	0.000442
Pfn1	947.83	1957.05	1.05	0.000122
Cdkn1a	158.06	329.32	1.05	0.000377
Gdnf	12.95	26.17	1.04	0.091634

Suppl.Table 3

mRNA	avg sh	avg str	log2FC	Padj
Gfap	337.06	2928.32	3.12	0.000191
Hmox1	32.35	242.43	2.91	1.01E-06
C1qc	41.97	262.46	2.64	1.22E-05
Tgfb1	20.38	118.75	2.54	0.001084
C1qa	38.47	224.20	2.54	0.000191
C1qb	65.80	362.41	2.46	0.013036
Cd44	30.82	159.37	2.37	0.000599
Ncf1	37.23	191.94	2.37	0.001115
Cd68	46.07	234.75	2.35	0.037777
Csf2rb	10.82	53.79	2.34	0.024867
Tnfrsf1b	25.24	127.96	2.33	0.003912
Fn1	73.62	367.47	2.32	0.060245
Fcrls	23.59	114.55	2.27	0.002458
Ccr2	14.73	71.13	2.27	0.022323
Nes	29.41	141.39	2.27	0.006308
Msn	28.70	132.50	2.21	0.00922
Itga5	19.86	92.11	2.20	0.022323
Trem2	34.50	154.24	2.16	0.001084
Tnfrsf1a	24.84	109.94	2.16	0.005658
Il4ra	38.55	164.61	2.09	0.005245
Itgam	37.63	154.85	2.04	0.00701
Psmb8	26.51	106.12	2.01	0.024867
Irf8	29.37	117.03	1.99	0.001084
Ccl12	10.32	39.30	1.96	0.026683
Tcirg1	80.20	298.46	1.90	0.067986
Lif	11.82	43.50	1.89	0.026683
Grn	69.67	242.72	1.80	0.063853
Sp100	20.36	67.93	1.74	0.061507
Il10ra	37.08	124.34	1.74	0.013036
Tspo	19.91	64.25	1.71	0.055405
Trf	575.98	1864.24	1.69	0.023845
Osmr	21.79	65.10	1.59	0.039337
Ccr5	34.44	103.34	1.58	0.028206
Cx3cr1	351.67	1042.06	1.57	0.024867
Nlrp3	15.75	44.19	1.51	0.041155
Ltbr	27.97	77.55	1.49	0.05714
Csf1r	223.47	621.06	1.47	0.026683
Casp8	18.59	50.85	1.45	0.062259
Spi1	23.91	62.24	1.40	0.063043
Cd9	44.38	115.45	1.39	0.045874
Il13ra1	21.80	54.77	1.35	0.028478
Tnfrsf12a	16.65	41.78	1.34	0.060245
Hexb	269.80	680.96	1.34	0.067986
Hspb1	36.56	91.40	1.33	0.026683
Csf1	82.64	207.29	1.32	0.004387
Hpgds	64.96	161.40	1.31	0.013036
Tgfbr2	52.52	129.24	1.30	0.048391
Tlr4	13.71	32.96	1.26	0.099062
Cd33	30.02	72.73	1.26	0.044327
Stab1	25.20	59.83	1.25	0.080157
Gusb	42.88	100.52	1.23	0.052605
Myc	72.28	165.01	1.18	0.033679
Stat1	80.47	181.63	1.17	0.03192
Cp	38.55	87.33	1.17	0.05714
Gsn	134.99	298.80	1.15	0.004387
Il6ra	40.85	89.43	1.13	0.084853
Il1r1	66.94	147.33	1.13	0.026683
Gjb1	86.33	188.23	1.13	0.022323
Stat3	130.44	274.51	1.07	0.00922
Tmem119	70.78	145.42	1.04	0.067986

Suppl. Fig. 1

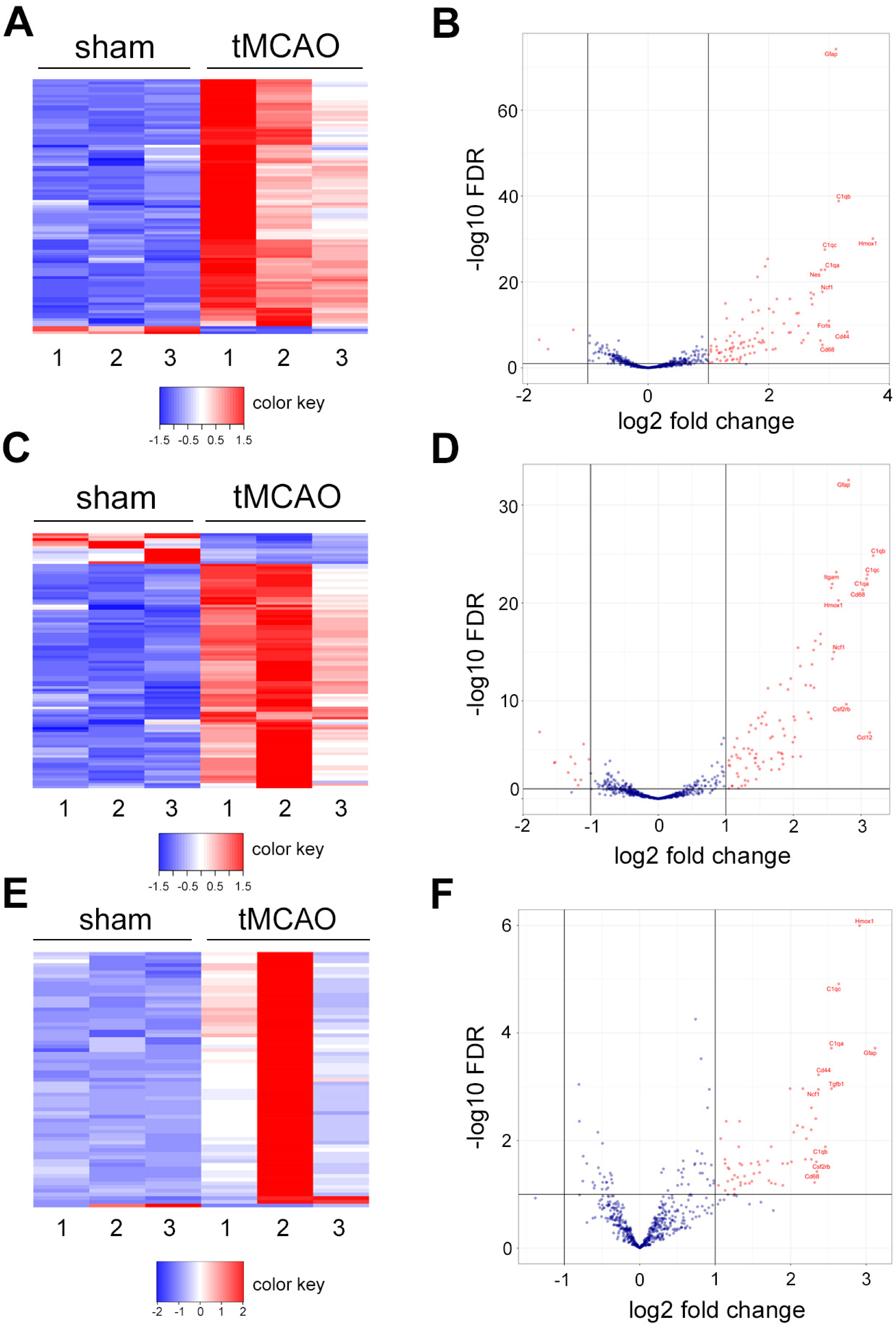

A
sham
tMCAO
1
2
3
1
2
3
color key
-1.5
-0.5
0.5
1.5
B
-log10 FDR
log2 fold change
Gfap
C1qb
Hmox1
C1qc
C1qa
Nes
Ncf1
Fcrls
Cd44
Cd68
C
sham
tMCAO
1
2
3
1
2
3
color key
-1.5
-0.5
0.5
1.5
D
-log10 FDR
log2 fold change
Gfap
C1qb
C1qc
Itgam
C1qa
Cd68
Hmox1
Ncf1
Csf2rb
Ccl12
E
sham
tMCAO
1
2
3
1
2
3
color key
-2
-1
0
1
2
F
-log10 FDR
log2 fold change
Hmox1
C1qc
C1qa
Gfap
Cd44
Tgfb1
Ncf1
C1qb
Csf2rb
Cd68

Suppl. Fig. 2

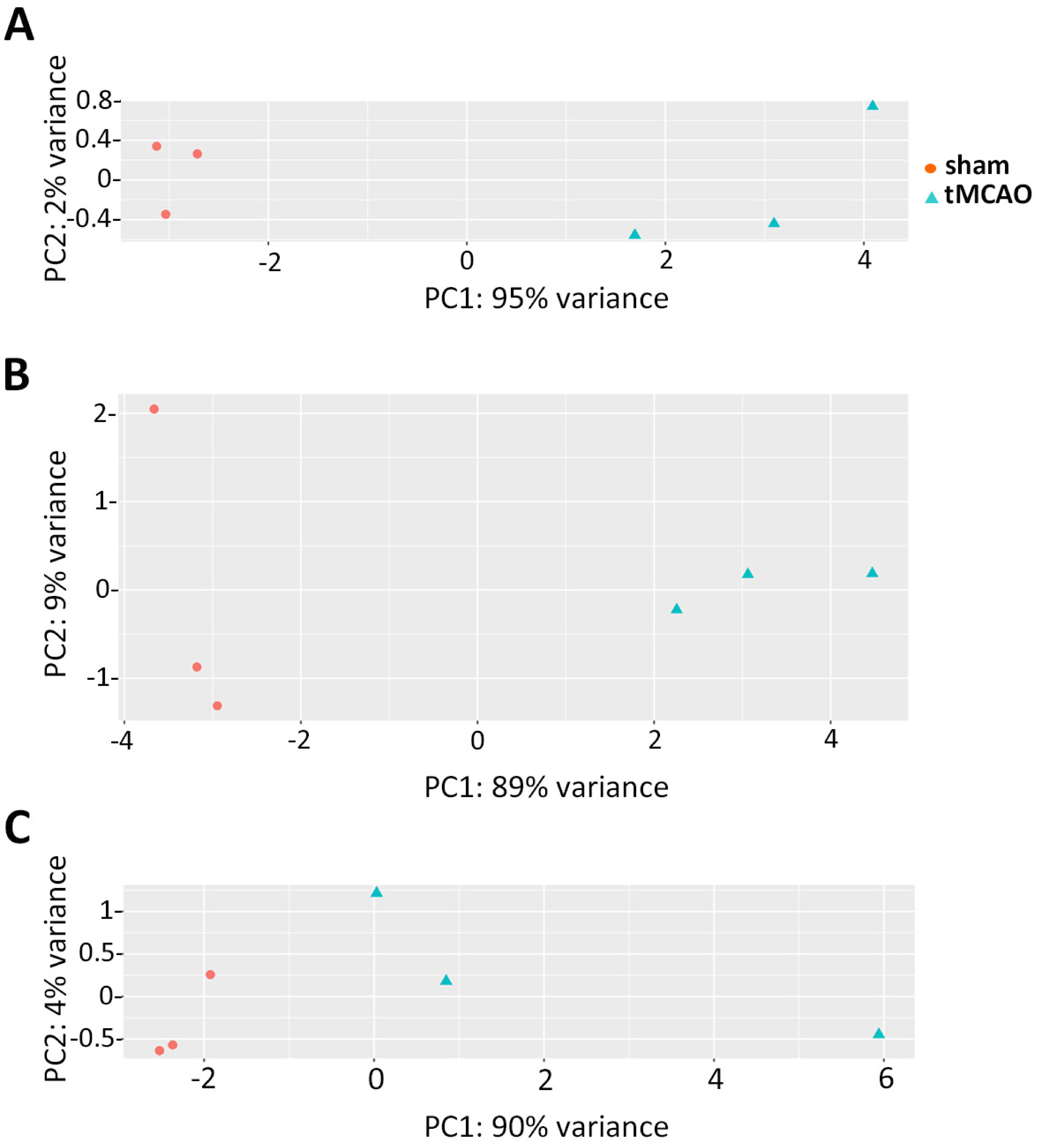
A
PC2: 2% variance
0.8
0.4
0
-0.4
-2
0
2
4
PC1: 95% variance
sham
tMCAO
B
PC2: 9% variance
2
1
0
-1
-4
-2
0
2
4
PC1: 89% variance
C
PC2: 4% variance
1
0.5
0
-0.5
-2
0
2
4
6
PC1: 90% variance

Suppl. Fig. 3

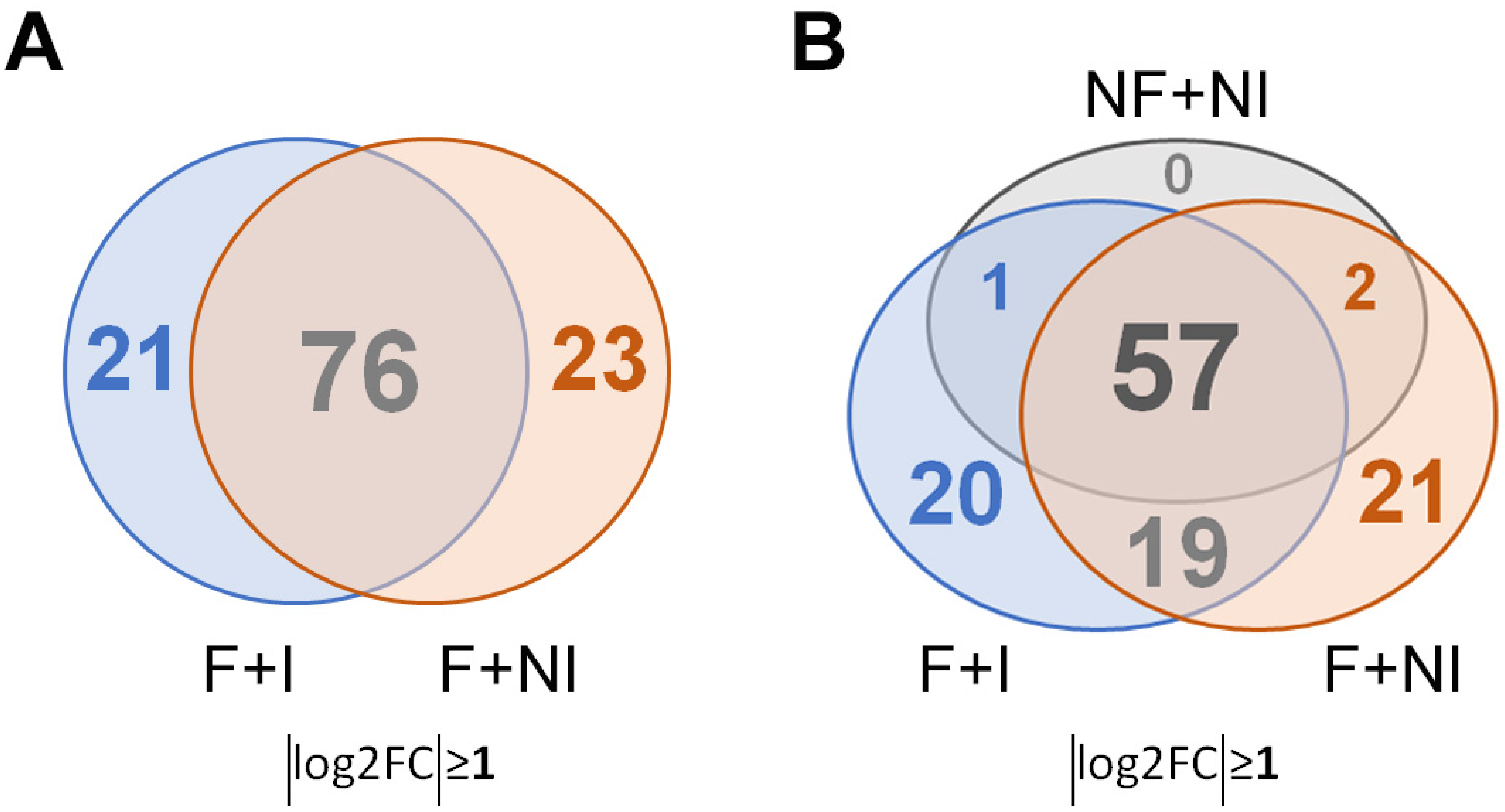

Suppl. Fig. 4

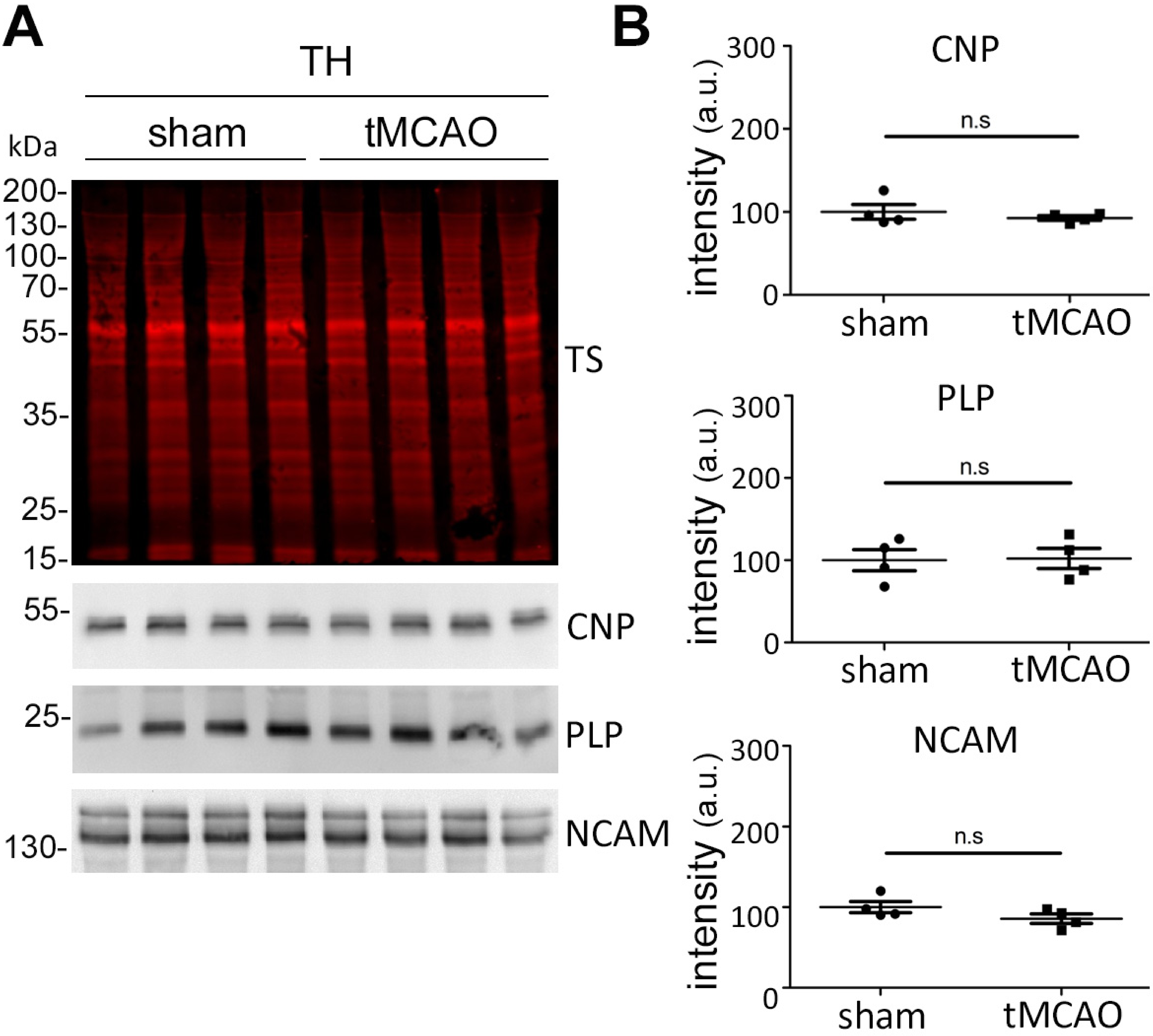

A
TH
sham
tMCAO
kDa
200-
130-
100-
70-
55-
35-
25-
15-
TS
55-
CNP
25-
PLP
130-
NCAM
B
CNP
PLP
NCAM
intensity (a.u.)
300
200
100
0
n.s
sham
tMCAO

Suppl. Fig. 5

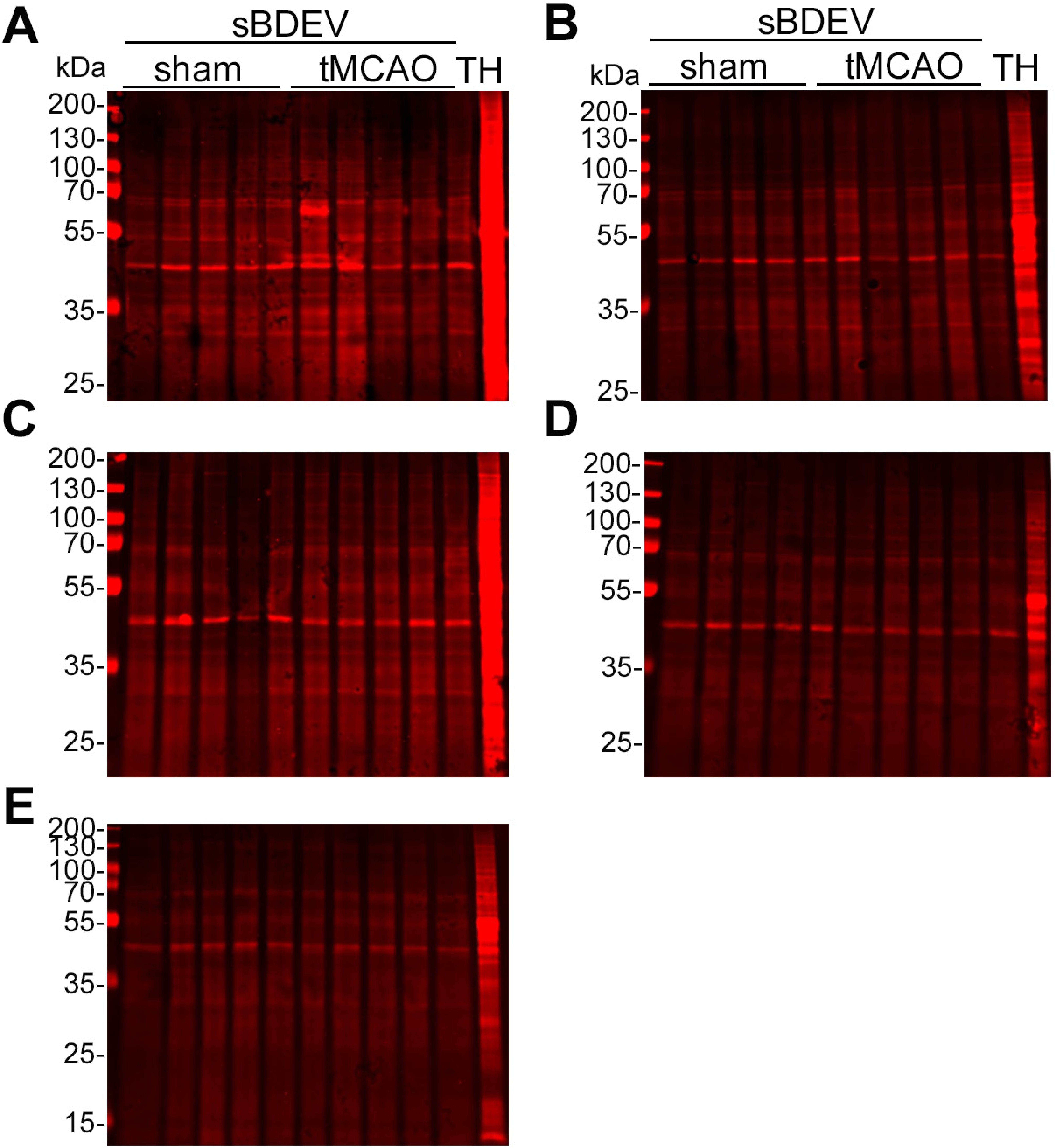
A
sBDEV
sham
tMCAO
TH
kDa
200-
130-
100-
70-
55-
35-
25-
B
sBDEV
sham
tMCAO
TH
kDa
200-
130-
100-
70-
55-
35-
25-
C
200-
130-
100-
70-
55-
35-
25-
D
200-
130-
100-
70-
55-
35-
25-
E
200-
130-
100-
70-
55-
35-
25-
15-
